# Anti-TNFα in inflammatory bowel disease: from originators to biosimilars

**DOI:** 10.3389/fphar.2024.1424606

**Published:** 2024-07-24

**Authors:** Zhen Zeng, Hao Lin, Mingshan Jiang, Jing Yuan, Xi Li, Yongbin Jia, Li Yang, Hu Zhang

**Affiliations:** ^1^ Department of Gastroenterology, West China Hospital, Sichuan University, Chengdu, China; ^2^ Centre for Inflammatory Bowel Disease, West China Hospital, Sichuan University, Chengdu, China; ^3^ Lab of Inflammatory Bowel Disease, Frontiers Science Center for Disease-Related Molecular Network, West China Hospital, Sichuan University, Chengdu, China; ^4^ General Practice Ward/International Medical Center Ward, General Practice Medical Center, West China Hospital, Sichuan University, Chengdu, China

**Keywords:** biosimilars, originators, anti-TNFα, biologics, inflammatory bowel disease

## Abstract

The introduction of anti-tumor necrosis factor α (TNFα) biologics significantly innovated inflammatory bowel disease (IBD) treatment and increased medical costs. The recent expiration of patents of some anti-TNFα biologics (such as infliximab and adalimumab) facilitated the development of biosimilars. Comparable pharmacokinetic, efficacy, safety, and immunogenicity profiles between anti-TNFα originators and biosimilars were demonstrated in different studies. Anti-TNFα biosimilars hold promise for reducing the high cost of biologics and increasing patient access to biologics. In this review, we outline the current data on the use of anti-TNFα originators and biosimilars in patients with IBD, with a focus on the efficacy, safety, and immunogenicity profiles of infliximab and adalimumab biosimilars. The potential benefits, challenges, and future directions of anti-TNFα biosimilars are also discussed in the review.

## 1 Introduction

**FIGURE 1:**
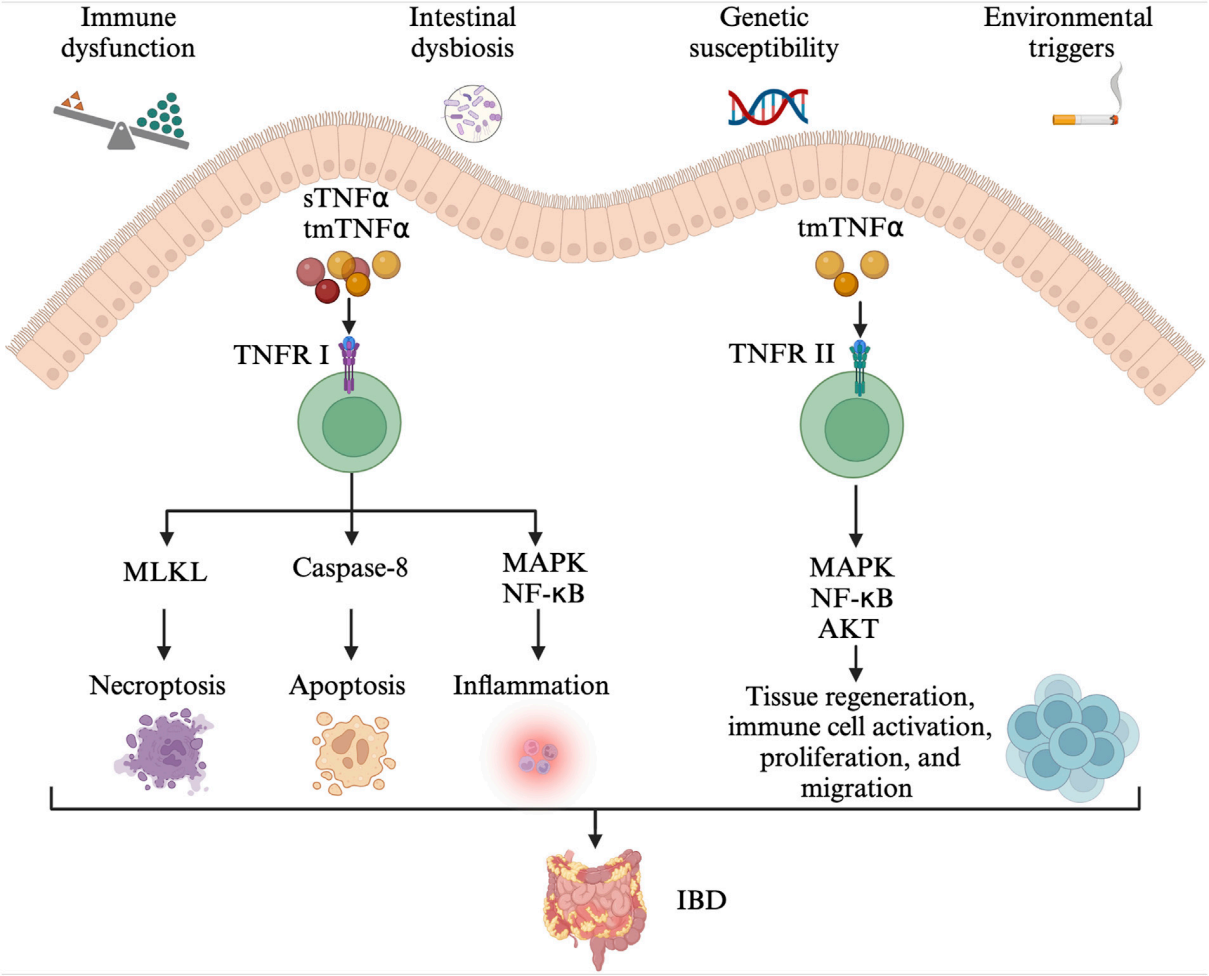
The pathogenesis of IBD. Immune dysfunction, intestinal dysbiosis, genetic susceptibility alongside environmental triggers contribute to the development of IBD. The tmTNFα and sTNFα bind with TNFRI, mediating the activation of pro-inflammatory (MAPK and NF-κB) signaling pathways, MLKL-dependent necroptosis, and caspase-8-dependent apoptosis. The TNFR2 signaling pathway is mainly activated by the tmTNF-α. The tmTNFα binds with TNFRII and activates MAPK, NF-κB, and AKT signaling pathways, involved in tissue regeneration, immune cell activation, migration, and proliferation. IBD: inflammatory bowel disease; tmTNFα: transmembrane TNFα; sTNFα: soluble TNFα; TNFRI: TNF receptor I; MAPK: mitogen-activated protein kinase; NF- NF-κB: nuclear factor kappa-B; MLKL: mixed-lineage kinase domain-like protein; TNFRII: TNF receptor II.

Inflammatory bowel disease (IBD) is a destructive, long-lasting, and immune-mediated disease, mainly including crohn’s disease (CD) and ulcerative colitis (UC) (Jiang et al., 2022). Despite significant advances have been made in exploring the occurrence and development of IBD, the exact pathogenesis is yet unclear. Immune dysfunction, intestinal dysbiosis, genetic susceptibility alongside environmental triggers may contribute to the development of IBD (Abraham and Cho, 2009; Dang et al., 2023) (Figure 1). It’s universally acknowledged that IBD is a global disease with high incidence and prevalence (Kaplan and Windsor, 2021). The chronic inflammation and remission-relapse pattern of IBD make patients experience chronic abdominal pain and repeated diarrhea, which exerts a significant impact on the quality of life (Chen et al., 2024). Available data indicated that the cumulative rates of hospitalization in CD and UC patients were 23%–49% and 9%–33% at 1 year; the 5-year hospitalization rates ranged between 44% to 54% and 18% to 54% for CD and UC, respectively. During the first 5 years after diagnosis, the cumulative rates for surgery were 5%–10% for UC and 10%–40% for CD. What should be noted is that the risk of developing colorectal cancer in patients with UC was two times higher than general population (Zhao et al., 2021). The high hospitalization and surgery rates, as well as high risk of developing cancers significantly increase medical costs for patients with IBD. In 2017, the global disability-adjusted life-years caused attributed to IBD was 1.85 million, about 1.5 times as that in 1990 (1.25 million) (GBD, 2017 Inflammatory Bowel Disease Collaborators, 2020). Indeed, it poses a huge burden on global healthcare systems.

Available data indicated that the mean healthcare costs for CD and UC were $8,265 and $5,066 per patient-year in the United States in 2004, respectively (Kappelman et al., 2008). From 2007 to 2016, the direct healthcare costs for IBD (CD and UC) increased to $22,987, three times higher than non-IBD controls ($6,956) (Park et al., 2020). In Europe, the mean total healthcare costs for CD and UC rose from €2,548 and €1,524 per patient-year in 2003 to €3,500 and €2,000 in 2020, respectively (Odes et al., 2006; Zhao et al., 2021). In China, the mean direct care costs for IBD (CD and UC) are $7,944 per patient-year from 2018 to 2019 (Yu et al., 2021). In the initial stages, the major drivers of healthcare costs for IBD were hospital and surgery. However, with the rapid progress made in drug development, the main health costs have shifted to medication. The global IBD medication treatment market size is extremely large. The introduction of biologics innovated IBD treatment and thus accounted for the majority of healthcare expenditures. Available data showed that biologics accounted for €1,782 for CD and €286 for UC per patient-year in Europe (Burisch et al., 2020). Anti-tumor necrosis factor-α (anti-TNFα) is the first approved biologic agent for CD and UC (Buchner et al., 2021). Among these biologics available for IBD, the annual costs of anti-TNFα treatment are considerable, making up 64% and 31% of the total costs in CD and UC, respectively (van der Valk et al., 2014). Although some anti-TNFα biologics have been included in medical insurance, the financial burden of IBD, especially for anti-TNFα biologic drugs, is still heavy. The high price further limits the access to anti-TNFα biologic treatment in resource-limited settings.

TNFα is a pro-inflammatory cytokine and plays an important role in the pathophysiology of IBD (Figure 1) (Chen L. et al., 2020; Chen et al., 2021). TNFα exists in two forms, the transmembrane and soluble form. On the one hand, transmembrane TNFα (tmTNFα) and the soluble TNFα (sTNFα) can bind with TNF receptor I (TNFRI), mediating the activation of mitogen-activated protein kinase (MAPK) and nuclear factor kappa-B (NF-κB) signaling pathways, and then, producing pro-inflammatory cytokines, cell adhesion molecules and synthetase nitric oxide (Wang and Shen, 2022). The binding between them also can activate caspase-8-dependent and mixed-lineage kinase domain-like protein (MLKL) death signaling pathways, involved in apoptosis and necroptosis, respectively (Jang et al., 2021). On the other hand, the binding of tmTNFα with TNF receptor II (TNFRII) can also activate MAPK, NF-κB, and AKT signaling pathways, causing tissue regeneration, immune cell activation, migration, and proliferation (Levin et al., 2016; Zeng et al., 2023). As a result, severe intestinal inflammation and mucosal barrier injury occur. In order to prevent its pro-inflammatory process, monoclonal antibodies to TNFα including infliximab, adalimumab, golimumab, and certolizumab have been developed and approved for CD and/or UC treatment (Leone et al., 2023). They may exert their therapeutic effects in the induction and maintenance of disease remission by inducing CD4^+^ T cell apoptosis and/or promoting the differentiation from monocytes to M2-type wound-healing macrophages (Levin et al., 2016).

Despite anti-TNFα biologics show favorable therapeutic effects in achieving clinical, endoscopic, and histologic remission in IBD, the annual costs are really high (Jiang et al., 2023). The expiration of patents of some anti-TNFα biologics has further facilitated the development of biosimilar agents. Biosimilars potentially reduce the high costs of biologics and increase patient access to biologics due to the stiff competition in the pharmaceutical market and extrapolation across indications (Fiorino and Danese, 2014). In this review, we briefly introduce the drug utilization, effectiveness, and safety of the most used anti-TNFα originators (infliximab and adalimumab), and elaborate on the efficacy and safety of these biosimilars in IBD. Furthermore, we also evaluate the efficacy and safety of the switches from originators to biosimilars, and discuss the benefits, challenges, and future directions of biosimilars in IBD.

## 2 The use of anti-TNFα originators in IBD

### 2.1 What are anti-TNFα originators

Anti-TNFα originators, discussed in this review, are the two anti-TNFα biologicals. Although biologicals comprise various groups of medicines, such as monoclonal antibodies, vaccines, growth factors, immune modulators, and medicines derived from human blood. Our review mainly discusses the two anti-TNFα monoclonal antibodies (infliximab and adalimumab). Anti-TNFα monoclonal antibodies are purified from human or mouse living systems, completely different from small molecules that are produced by chemical synthesis or purified from plants (Buchner et al., 2021). Anti-TNFα originators are a diverse group of original, independent research and development new drugs with pharmaceutical patents, usually used as licensed reference products (Kang et al., 2023). Anti-TNFα originators follow a complex and long process for regulatory approval, including drug screening and optimization (structure, pharmacologic action, and biological activity), preclinical studies (pharmacokinetics, pharmacodynamics, and toxicology *in vitro* and *in vivo* studies), clinical studies (I–III randomized clinical trials), marketing approval, and post-marketing research (IV clinical trial), which significantly increases the time and money costs. Besides, the manufacturing costs of anti-TNFα originators are very high. Available data indicated that the cost to develop a new biological agent is about $2.0 billion, significantly higher than the production costs of biosimilars ($100–250 million) (Zheng et al., 2017).

### 2.2 Infliximab

Infliximab, a human-mouse chimeric anti-TNFα monoclonal IgG1 antibody, is the first biologic approved for CD by the United States Food and Drug Administration (FDA) in 1998. It binds with TNFα and prevents the binding between TNFα and TNFR (Knight et al., 1993). Until now, it has been approved for various indications including CD, UC, rheumatoid arthritis (RA), psoriasis, and others. The famous ACCENT I randomized trial of 573 moderate to severe CD patients showed that infliximab can induce disease response at week 2 in 58% (335/573) of patients. At week 30, the clinical remission rates were higher in the infliximab maintenance group (5 mg/kg infliximab and 10 mg/kg infliximab), compared with the placebo group (39% vs. 21%, 45% vs. 21%, respectively). The maintenance treatment efficacy of infliximab was also claimed at week 54. At week 54, the proportion of patients who discontinued corticosteroid treatment in the infliximab maintenance group was 2.22 times higher than that of the placebo group (29% vs. 9%). Besides, patients in the infliximab maintenance group also presented lower mean Crohn’s Disease Activity Index (CDAI) and higher mean inflammatory bowel disease questionnaire (IBDQ) scores (Hanauer et al., 2002). Recently, a network meta-analysis of 25 clinical trials and 8,720 CD patients claimed that infliximab had optimal efficacy in the induction of clinical remission in patients with luminal CD (Barberio et al., 2023). The excellent therapeutic effects of infliximab were also confirmed in another ACCENT II trial of 306 fistulizing CD patients. In comparison with the placebo group (19%), 36% of patients with infliximab treatment had a total absence of fistulas at week 54 (Sands et al., 2004). As for the safety of infliximab, the FDA label indicated that the risk of serious adverse events (SAEs) including serious infections and malignancy increased in the infliximab treatment group, although SAE rates were similar between the infliximab treatment arm and the placebo arm in another study (Sands et al., 2004; Food and Drug Administration, 2021). In a word, infliximab treatment is effective and safe in inducing and maintaining disease remission in moderate to severe CD.

In UC, the Active Ulcerative Colitis Trials 1 and 2 (ACT 1 and ACT 2) of 364 moderate to severe UC patients revealed that infliximab therapy can induce clinical remission and mucosal healing as early as week 8, and maintain effective during week 54 (Rutgeerts et al., 2005). As for patients with steroid-refractory acute severe ulcerative colitis (ASUC), infliximab outperformed cyclosporine in achieving endoscopic remission at day 98 (73% vs. 25%) (Laharie et al., 2021). Moreover, infliximab was also claimed to be an effective salvage treatment for patients with tacrolimus-refractory ASUC (Yamamoto et al., 2010). It was also proven to be safe in treating UC with regard to similar rates of adverse events (AEs), infections, and acute infusion reactions (Rutgeerts et al., 2005). It should be noted that infliximab is a chimeric antibody, implying a higher possibility of formation of antibodies to infliximab. As a result, the risk of experiencing infusion reactions and even loss of efficacy may increase (Su and Lichtenstein, 2003). Concomitant immunosuppressive therapy or changing antibody structure may mitigate immunogenic responses (Su and Lichtenstein, 2003). Indeed, the introduction of infliximab innovated IBD therapy and became the mainstay of treatment for refractory IBD. Further studies on other anti-TNFα biologics are therefore encouraged.

### 2.3 Adalimumab

Adalimumab is also an anti-TNFα monoclonal IgG1 antibody, but it is different from infliximab regarding antibody structure (Tracey et al., 2008). It is a fully human, recombinant monoclonal antibody with lower immunogenicity and a larger antigen-antibody interface (Tracey et al., 2008; Hu et al., 2013; Kennedy et al., 2019). The CLASSIC-I trial of 299 moderate to severe CD patients (naive to anti-TNFα antagonists) claimed that the adalimumab 160/80 treatment (160 mg at week 0 and 80 mg at week 2) was more effective than placebo in inducing clinical remission (36% vs. 12%) (Hanauer et al., 2006). One year later, the CLASSIC II trial further demonstrated its significant efficacy and safety in maintaining clinical remission during week 56. In comparison with the placebo group, the adalimumab treatment group (40 mg every other week) presented higher remission rates (79% vs. 44%), greater mean decreases of CDAI scores (197.7 vs. 119.6), and higher IBDQ scores (Sandborn et al., 2007). In the same year, the better therapeutic effects of adalimumab were also found in those CD patients previously exposed to anti-TNFα therapy. This finding suggested that adalimumab could be an additional treatment option for those who lost response to and/or were intolerant to infliximab. Besides, patients receiving adalimumab were more likely to achieve corticosteroid-free remission and fistula remission than the placebo group (Colombel et al., 2007). Recently, the CREOLE study further evaluated the efficacy of adalimumab in CD patients with symptomatic small bowel stricture (SSBS) and proved its excellent effects in patients with SSBS due to CD. Treatment with adalimumab can make 53% of patients free of surgery 4 years after initiation (Bouhnik et al., 2018). A large meta-analysis of 31 clinical trials recommended adalimumab as second-line therapy for patients who were intolerant to infliximab (Singh et al., 2021).

As for moderate to severe UC patients, a multicenter study of 576 patients suggested that the clinical remission rates at week 8 in the adalimumab subcutaneous injection regimen arm (160 mg at week 0 and 80 mg at week 2) were one time higher than that in the placebo group (18.5% vs. 9.2%) (Reinisch et al., 2011). At week 52, 17.3% of patients in the adalimumab group maintained clinical remission, compared with 8.5% of patients in the placebo group. Moreover, more patients with adalimumab therapy achieved sustained mucosal healing (at week 8 and week 52), and sustained corticosteroid-free remission (at week 32 and 52) than the placebo group (18.5% vs. 10.6%, and 10.0% vs. 1.4%, respectively) (Sandborn et al., 2012). Even in those patients with a history of anti-TNFα therapy, the adalimumab treatment group was more likely to maintain sustained clinical response at week 8, and week 52 than the placebo group, providing an alternative therapeutic option to patients who experienced infliximab failure (Sandborn et al., 2012). A cost-effectiveness analysis from the United Kingdom further claimed that the total costs (including costs of drug acquisition and administration, direct and indirect healthcare costs) and biologic costs for adalimumab was lower than infliximab (£194,764.73 vs. £206,065.90, and £10,289.40 vs. £19,285.37, respectively). And patients on adalimumab incurred slightly higher quality-adjusted life years (QALYs) than those on infliximab treatment (13.872 vs. 13.788) (Wilson et al., 2018). Caution needs to be taken when interpreting these results, because different study designs, different standards of treatment response, and different ethnicities are used in various studies. Collectively, adalimumab is an effective and well-tolerated biologic drug for moderate to severe CD and UC patients. It also became the efficacy benchmark of its category and the reference product for bioequivalence studies.

## 3 The use of anti-TNFα biosimilars in IBD

### 3.1 What are anti-TNFα biosimilars

**FIGURE 2:**
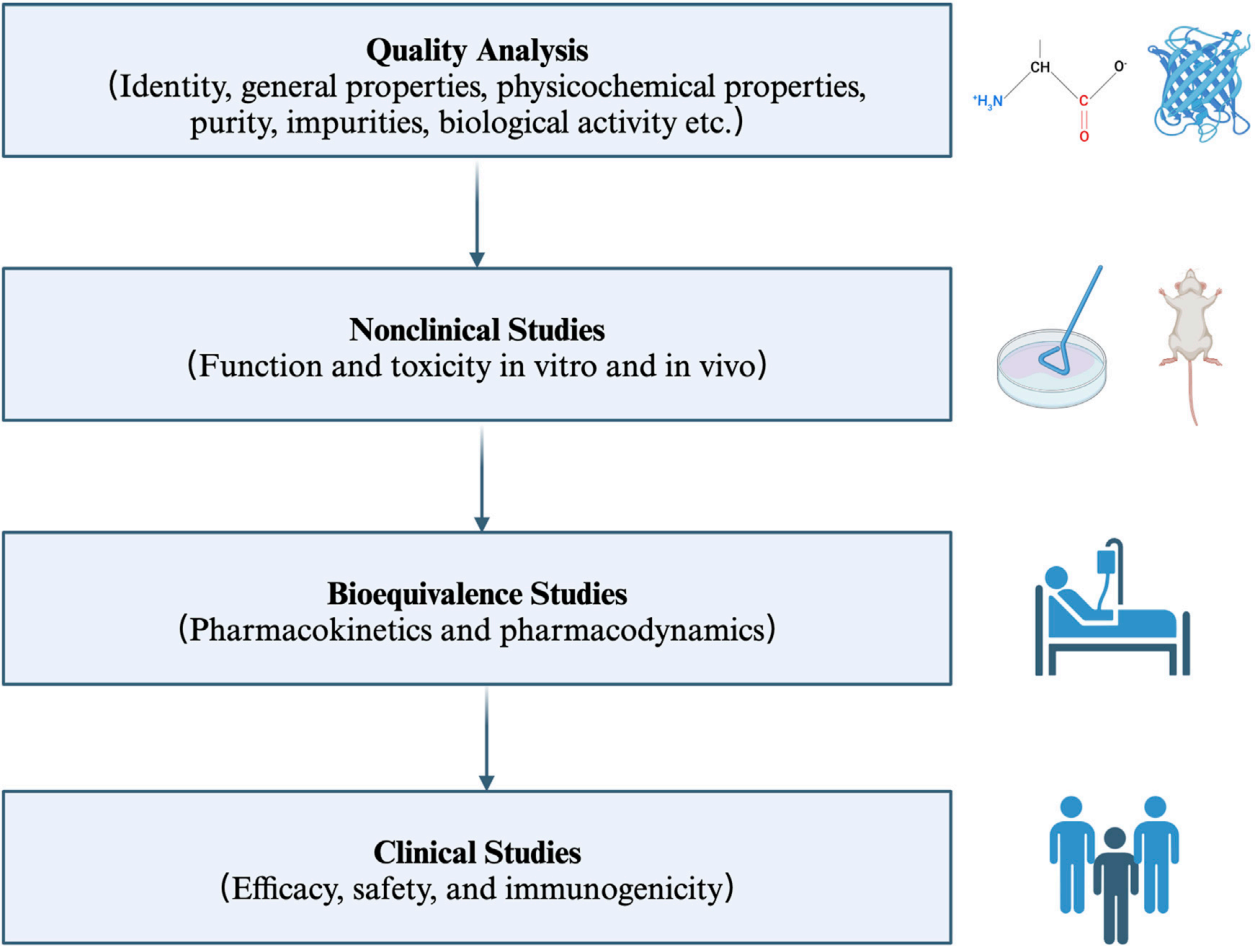
Key principles for the licensing of biosimilars by WHO. The regulatory and approval pathway for biosimilars includes four steps. Firstly, characterize the quality attributes of the biosimilar and the reference product. Secondly, evaluate the pharmaco-toxicological activity of the biosimilar and the reference product *in vitro* and *in vivo*; Thirdly, investigate the pharmacokinetic and pharmacodynamic profiles in healthy volunteers; Fourthly, assess the efficacy, safety, and immunogenicity profiles in one or more indications. WHO: World Health Organization.

Biosimilars are biological products that are similar in terms of quality, safety, and efficacy to an already licensed reference product (World Health Organization, 2022). Therefore, the anti-TNFα biosimilars are a group of monoclonal antibodies that contain a version of the active pharmaceutical ingredient and associated molecules of already licensed original biologics (originators) (Blandizzi et al., 2017; World Health Organization, 2022). Anti-TNFα biosimilars are different from generic medicines in terms of the drug substance. The former contains similar active ingredients, while the latter has identical active ingredients (Blandizzi et al., 2017). Moreover, given the relatively high molecular weight, complicated three-dimensional protein structure, and complex posttranslational modification, the structural sameness and bioequivalence evaluation approaches used in generic medicines is not applicable to biosimilars. Firstly, researchers should characterize the quality attributes of the reference product, and make direct head-to-head comparison between the licensed reference product and the biosimilar in terms of structural and functional similarity (*in vivo* and *in vitro*). Then, the clinical pharmacologic comparability assessment (pharmacokinetic modeling, pharmacodynamic modeling and immunogenicity) is carried out in one or more indications (if possible). Then, the comparative clinical trials (safety, efficacy, and immunogenicity profiles) are performed in one or more sensitive populations (if possible) (Lyman et al., 2018; World Health Organization, 2022) (Figure 2).

The regulatory process for approval of anti-TNFα biosimilars is speedier and easier in comparison with originators. The core evidence to support regulatory approval for anti-TNFα biosimilars is obtained from manufacturing and preclinical data. While the marketing approval for originators depends more on extensive clinical data. Besides, extrapolation across indications further accelerates the regulatory approval process of anti-TNFα biosimilars. Once clinical bioequivalence is fulfilled in one condition, this biosimilar may be approved for other indications for which the reference product has been approved, without the need for repeating clinical trials across different indications. This process is called extrapolation (Lyman et al., 2018). Most clinical equivalence studies of anti-TNFα biosimilars have been conducted in patients with RA and/or those with plaque psoriasis, rarely in patients with IBD (Ben-Horin et al., 2016). Collectively, anti-TNFα biosimilars follow an accelerated process for marketing approval.

The biosimilar, Omnitrope, was approved by the European Medicines Agency (EMA) for patients with growth hormone deficiency in April 2006 (Fuhr et al., 2010). It is the first biosimilar approved for patients. Seven years later, biosimilars of infliximab, Remsima and Inflectra (CT-P13) got approval for CD or UC in September 2013. They two have become the first monoclonal antibody biosimilar approved by the EMA. In 2016, Inflectra was firstly approved by the FDA for patients with IBD. One year later, the biosimilar of adalimumab, Amjevita (ABP 501) was approved for patients with IBD. In recent 10 years, several other biosimilars of infliximab such as Flixabi (SB2) and Zessly (PF-06438179/GP1111) (Table 1), and biosimilars of adalimumab including Imraldi (SB5), Cyltezo (BI 695501), Halimatoz (GP 2017), and Idacio (MSB11022) have been approved for patients with IBD, significantly expanding the treatment options for patients (Generics and Biosimilars Initiative, 2023a; Generics and Biosimilars Initiative, 2023b) (Table 2). In this part, we mainly discuss the most studied biosimilars of infliximab (Supplementary Table S1) and biosimilars of adalimumab in IBD (Supplementary Table S2).

**TABLE 1:** Approval status of infliximab biosimilars.

Originator	INN	Trade name	Manufacturer name	Approval status
Infliximab	ABP 710	Avsola	Amgen, United States	FDA: December 2019; Canada: March 2020
Infliximab	BOW015	Infimab	Epirus Biopharmaceuticals, United States	India: September 2014
Infliximab	CMAB008	Ting Lei	Mabpharm, China	NMPA: July 2021
Infliximab	CT-P13	Remsima	Celltrion, South Korea	EMA: September 2013 (IV), September 2019 (SC); Japan: July 2014; South Korea: July 2012; Canada: January 2014 (IV), January 2021 (SC)
Infliximab	CT-P13	Inflectra	Pfizer (Hospira), United States	FDA: April 2016; Canada: January 2014; Australia: August 2015
Infliximab	CT-P13	Saixi	Celltrion, South Korea	NMPA: June 2023
Infliximab	CT-P13	Flammegis	Celltrion, South Korea	Russia: July 2015
Infliximab	CT-P13	Infliximab biosimilar 1	Celltrion, South Korea/Nippon Kayaku, Japan	Japan: July 2014
Infliximab	GB242	Jian Jiayou	Genor Biopharma, China	NMPA: February 2022
Infliximab	HS626	Baite An	BioRay, China	NMPA: September 2021
Infliximab	N/A	Infliximab biosimilar 3	Pfizer Japan	Japan: July 2018
Infliximab	NI-071	Infliximab biosimilar 2	Nichi-Iko Pharmaceutical, Japan	Japan: September 2017
Infliximab	PF-06438179	Ixifi	Pfizer, United States	FDA: December 2017; Canada: December 2021
Infliximab	PF-06438179/	Zessly	Sandoz, Switzerland	EMA: May 2018
Infliximab	SB2	Flixabi	Samsung Bioepis, South Korea	EMA: May 2016
Infliximab	SB2	Renflexis	Samsung Bioepis, South Korea; Merck, United States	FDA: April 2017; South Korea: December 2015; Australia: November 2016, Canada: December 2017

Abbreviations: INN, International non-proprietary names; EMA, European Medicines Agency; IV, intravenous; SC, subcutaneous; FDA, Food and Drug Administration; NMPA, National Medical Products Administration.

**TABLE 2:** Approval status of adalimumab biosimilars.

Originator	INN	Trade name	Manufacturer name	Approval status
Adalimumab	ABP 501	Amjevita	Amgen, United States	FDA: September 2016
Adalimumab	ABP 501	Amgevita	Amgen, United States	EMA: 21 March 2017; Canada: November 2020; Australia: October 2017
Adalimumab	ABP 501	Solymbic	Amgen, United States	EMA: March 2017, withdrawn on March 2019
Adalimumab	ABP 501	Adalimumab biosimilar 2	Daiichi Sankyo, Japan/Amgen, United States	Japan: January 2021
Adalimumab	AVT02	Hukyndra	Alvotech, Iceland/Stada Artnimettel, Germany	EMA: November 2021
Adalimumab	AVT02	Libmyris	Alvotech, Iceland/Stada Artnimettel, Germany	EMA: November 2021
Adalimumab	AVT02	Simlandi	Alvotech, Iceland/Teva, Israel	FDA: February 2024; Canada: January 2022
Adalimumab	BAT1406	QLETLI	Bio-Thera, China	NMPA: October 2019
Adalimumab	BCD-057	Dalibra	Biocad, Russia	Russia: February 2019
Adalimumab	BI 695501	Cyltezo	Boehringer Ingelheim, Germany	FDA: August 2017; EMA: November 2017, withdrawn on January 2019
Adalimumab	CHS-1420	Yusimry	Coherus Biosciences, United States	FDA: December 2021
Adalimumab	CT-P17	Yuflyma	Celltrion, South Korea	EMA: February 2021; Canada: December 2021
Adalimumab	FKB327	Hulio	Mylan/Fujifilm Kyowa Kirin Biologics, United States	FDA: July 2020; EMA: September 2018; Canada: November 2020; Japan: June 2020
Adalimumab	GP2017	Hyrimoz	Sandoz, Switzerland	FDA: October 2018; EMA: July 2018; Canada: November 2020
Adalimumab	GP2017	Halimatoz	Sandoz, Switzerland	EMA: July 2018, withdrawn on January 2021
Adalimumab	GP2017	Hefiya	Sandoz, Switzerland	EMA: July 2018
Adalimumab	HLX03	Yuan Handa	Shanghai Henlius Biotech, China	NMPA: December 2020
Adalimumab	HS 016	Jianning An	BioRay, China	NMPA: December 2019
Adalimumab	IBI-303	Sulinno	Innovent, China	NMPA: September 2020
Adalimumab	LBAL	adalimumab biosimilar 3	LG Life Sciences, South Korea; Mochida Pharmaceutica, Japan	Japan: March 2021
Adalimumab	MSB11022	Idacio	Fresenius Kabi, Germany	FDA: December 2022; EMA: April 2019; Canada: October 2020
Adalimumab	MSB11022	Kromeya	Fresenius Kabi, Germany	EMA: April 2019, withdrawn on December 2019
Adalimumab	N/A	Mabura	Hetero Drugs, India	India: January 2018
Adalimumab	N/A	Adfrar	Torrent Pharmaceuticals, India	India: January 2016
Adalimumab	N/A	Cadalimab	Zydus Cadila, India	India: August 2020
Adalimumab	PF-06410293	Abrilada	Pfizer, United States	FDA: November 2019; Canada: June 2021
Adalimumab	PF-06410293	Amsparity	Pfizer, United States	EMA: February 2020
Adalimumab	SB5	Hadlima	Samsung Bioepis, South Korea	FDA: July 2019; Canada: May 2018; Australia: January 2018; South Korea: September 2017
Adalimumab	SB5	Imraldi	Samsung Bioepis, South Korea	EMA: August 2017
Adalimumab	SCT630	Jianrun An	SinoCellTech, China	NMPA: June 2023
Adalimumab	TQ-Z2301	Bowei Tai	Chiatai Tianqing, China	NMPA: January 2022
Adalimumab	UBP1211	Maikang Jun	Shanghai Junshi Biosciences, China	NMPA: March 2022
Adalimumab	ZRC3197	Exemptia	Zydus Cadila, India	India: September 2014

Abbreviations: INN, International non-proprietary names; FDA, Food and Drug Administration; EMA, European Medicines Agency; NMPA, National Medical Products Administration.

### 3.2 Biosimilars of infliximab in IBD

#### 3.2.1 CT-P13

CT-P13 is the first approved biosimilar of infliximab used in immune-mediated diseases including RA, ankylosing spondylitis (AS), psoriasis, CD, and UC (Parigi et al., 2021). Two clinical head-to-head studies, the PLANETRA study, and PLANETAS study, demonstrated that CT-P13 (intravenous, IV formulation) was non-inferior to infliximab originator in terms of clinical efficacy and safety. Moreover, comparable pharmacokinetic and immunogenicity profiles have also been claimed in the two studies (Park et al., 2013; Yoo et al., 2013). Therefore, CT-P13 was approved for CD and UC based on extrapolation. The PROSIT-BIO cohort study of 547 patients with IBD firstly suggested that 73.7% of anti-TNF naïve patients with CT-P13 treatment could achieve clinical response at week 24, which was comparable with infliximab therapy (Fiorino et al., 2017). Following head-to-head comparison between infliximab and CT-P13 was conducted in 220 patients with active CD. The week 6 clinical response rates (a decrease of 70 points or more in CDAI, CDAI-70) were similar between the infliximab treatment group and the CT-P13 therapy group (74.3% vs. 69.4%). Furthermore, the two groups also showed comparable clinical remission rates and steroid-free remission rates at week 30. No significant differences in mean C reactive protein (CRP) concentrations, mean fecal calprotectin (FC) levels, pharmacokinetic, and pharmacodynamic profiles (C_max_ and C_trough_) were observed between the two groups at every visit (Ye et al., 2019). Another comparative equivalence cohort study of 5050 infliximab naïve CD patients further proved the therapeutic equivalence between CT-P13 and infliximab (Meyer et al., 2019). Recently, a subcutaneous (SC) formulation of CT-P13 (CT-P13 SC) was developed for immune-mediated diseases. Although CT-P13 SC has its inherent shortcomings in comparison with the IV formulation of CT-P13 (CT-P13 IV), such as slower absorption, inadequate bioavailability, and lower initial peak concentrations, it shows its superiority in convenience, easy access, and time-saving (Bittner et al., 2018). It was claimed to be non-inferior to CT-P13 IV in patients with RA and IBD (Reinisch et al., 2019; Schreiber et al., 2021). An open-label, randomized, phase 1 study of 53 active CD and 78 active UC evaluated the efficacy and safety profiles of CT-P13 SC and suggested that the clinical response rates (86.8% vs. 74.4%, at week 30), clinical remission rates (60.5% vs. 38.5%, at week 30), and mucosal healing rates (47.7% vs. 30.8%, at week 22) were not significantly different between the CT-P13 SC group and the CT-P13 IV group. Besides, no differences in safety (treatment-emergent adverse events, TEAEs, 57.6% vs. 49.2%) and pharmacokinetics (C_trough_ 21.45 μg/mL vs. C_trough_ 2.93 μg/mL, at week 22) were found between the two arms, despite CT-P13 SC group showed numerically higher C_trough_ during week 6 to week 54 (Schreiber et al., 2021). CT-P13 SC indeed provides an additional alternative for IBD patients. It holds the promise of reducing medical visit-associated costs, optimizing medical resources, and reducing the burden on the healthcare systems. It is also an important step towards patient empowerment and medication self-management in IBD treatment.

What should be noted is that switching from originator infliximab to biosimilar CT-P13 was also claimed to be safe and tolerated (Schmitz et al., 2018; Ye et al., 2019; Haifer et al., 2021). At week 54, the clinical response rates and clinical remission rates were similar between the continued treatment group and the switching treatment group (Ye et al., 2019). These results were in accord with similar findings by the pivotal NOR-SWITCH study that switching from infliximab to CT-P13 IV was not inferior to continued treatment with infliximab in patients with immune-mediated diseases including CD, UC, RA, and others (Jørgensen et al., 2017). The two groups presented similar disease worsening rates during 54-week follow-up (26% vs. 30%). Moreover, they also claimed no notable differences in trough drug concentrations in the two groups (Jørgensen et al., 2017). Comparable serum drug concentrations between the maintenance and the switching treatment group were also demonstrated in the SECURE study (Strik et al., 2018). Switching from originator infliximab to CT-P13 SC is also safe and tolerated (Smith et al., 2022). Concerns regarding safety and immunogenicity arose when we made a non-medical switch from originators to biosimilars. The NOR-SWITCH extension study of 380 patients with immune-mediated disease revealed that treatment switching did not increase the incidence of anti-drug antibodies (ADAbs) and AEs during 78-week follow-up (Goll et al., 2019). Several studies also demonstrated no differences in safety and immunogenicity between the maintenance and the switching treatment group (Jørgensen et al., 2017; Strik et al., 2018; Meyer et al., 2019; Ye et al., 2019). However, a contrary result that CT-P13 was inferior to infliximab was showed in another study (Chaparro et al., 2019). Chaparro et al. (Chaparro et al., 2019) claimed that switching treatment increased the risk of disease relapse in patients with IBD. Cautions need to be made when we interpret these results. Various definitions of disease relapse, disease remission, clinical remission, and disease worse were set in different studies. What’s more, the time for switching treatment from originators to biosimilars was also different, bringing additional hurdles to explain these findings. Further studies should be taken to elucidate these uncertainties.

#### 3.2.2 SB2

SB2 is the second infliximab biosimilar approved for CD and UC. One phase I study and another phase III clinical trial demonstrated its equivalence of pharmacokinetics, efficacy, and safety with originator infliximab in healthy volunteers and patients with RA, paving the way to SB2 approval in RA and other immune-mediated diseases (Shin et al., 2015; Choe et al., 2017). As for IBD, a prospective observational study assessed its efficacy and safety in 276 patients with IBD (136 CD and 140 UC). 57.3% of infliximab naïve patients can achieve steroid-free remission after an 8-week SB2 treatment, which is similar to the effectiveness of infliximab and CT-P13 (Macaluso et al., 2021b). One aspect should be taken into consideration is that previous anti-TNF treatment may decrease the efficacy of SB2 in IBD. In comparison with anti-TNF-naïve cases, patients who were previously exposed to anti-TNF presented lower steroid-free remission rates (66.1% vs. 40.0%) (Macaluso et al., 2021b). Another real-life study of 85 patients with IBD further verified its efficacy and immunogenicity. No significant differences in clinical remission rates, FC levels, and corticosteroid-free rates have been found after switching from infliximab to SB2 treatment (at a mean time of 329 days). Switching treatment also did not increase the risk of developing ADAbs and SAEs during a mean 135-day follow-up (Massimi et al., 2021). The long-term effectiveness, safety, and immunogenicity were further investigated by a German research. During an 80-week follow-up, the changes in the Harvey-Bradshaw Index (HBI) and partial Mayo Score (PMS) were not significant after switching treatment. Furthermore, about 72% of patients persisted in SB2 therapy at week 78, indicating that this switch was well tolerated (Fischer et al., 2021). The safety profile of SB2 in IBD varies between different studies. Some studies did not record any SAEs, while some other studies claimed that about 7.6%–20.7% of patients might suffer from SAEs (Fiorino et al., 2017; Fischer et al., 2021; Massimi et al., 2021; Bouhnik et al., 2023). The inconsistency in follow-up time might partly explain the difference in SAEs.

A single switch from infliximab to SB2 is claimed to be safe and tolerated in patients with IBD. Multiple switches from originators to CT-P13 to SB2 are still demonstrated to be safe and effective. An observational study evaluated the effects and pharmacokinetics of the first switch (from CT-P13 to SB2) and the second switch (from infliximab to CT-P13 to SB2) in 186 patients with IBD. No significant changes in CRP, HBI, or Simple Clinical Colitis Activity Index were found upon the first and second switches. Similar median C_trough_ was recorded in pre-switch, early, and 1-year post-switch (4.9 μg/mL vs. 5.5 μg/mL vs. 5.3 μg/mL). Moreover, switching treatment did not exert a negative influence on disease response, given the comparable response rates during the 1-year follow-up (91% vs. 92% vs. 95%) (Luber et al., 2021). Another prospective multicenter cohort study of 176 patients with IBD further provided convincing evidence of efficacy and safety for multiple switches from originators to different biosimilars. The first switch (from CT-P13 to SB2) and the second switch (from infliximab to CT-P13 to SB2) showed comparable clinical remission rates at 12 months after switching treatment. Besides, 62.5% of the first switch group and 72.2% of the second switch group presented low FC levels (<250 mg/kg). It is worth noting that only the first switch group reported infusion reactions (3/80, 3.8%), suggesting multiple switches did not increase the risk of AEs (Hanzel et al., 2022). As aforementioned, the risk of increased immunogenicity is one of the core concerns when we make multiple switches. Available data demonstrated that no new ADAbs were developed after multiple switches (Hanzel et al., 2022). Although SB2 was claimed to be safe and effective in several studies, the clinical equivalence of SB2 in IBD is mostly proven in real-world studies, indicating a pressing need to conduct randomized, head-to-head, parallel clinical trials. Furthermore, few studies evaluated the efficacy of SB2 in achieving higher therapeutic goals, such as endoscopic mucosal healing and histologic remission. More studies are warranted to fill this gap.

#### 3.2.3 PF-06438179/GP1111

PF-06438179/GP1111 is another biosimilar of infliximab, which was approved for immune-mediated diseases by the FDA in 2017 and by the EMA in 2018 (Generics and Biosimilars Initiative, 2023a; Generics and Biosimilars Initiative, 2023b). One phase I clinical study evaluated the pharmacokinetics and immunogenicity of PF-06438179/GP1111 in 151 healthy subjects. The PF-06438179/GP1111 group showed great similarities in serum concentration-time profiles and ADAb response rates to the infliximab group (Palaparthy et al., 2018). The equivalent safety and efficacy of PF-06438179/GP1111 was demonstrated in a large randomized controlled trial of 650 patients with moderate to severe active RA (Cohen et al., 2018b). There are no significant differences in the American College of Rheumatology (ACR)-20, ACR-50, and ACR-70 response rates between the PF-06438179/GP1111 treatment group and the infliximab treatment group. Comparable Disease Activity Score (DAS) remission rates, and the 2010 American College of Rheumatology/European League Against Rheumatism (ACR/EULAR) remission rates were also claimed in this study. Besides, the PF-06438179/GP1111 arm showed similar all-cause TEAEs, incidence of ADAbs, and median C_trough_ concentrations to the infliximab arm. When we made a non-medical switch from originator infliximab to PF-06438179/GP1111, efficacy was also well sustained in terms of ACR20, ACR50 and ACR70 response rates (Cohen et al., 2018b). This result indicated that single switch from a originator to PF-06438179/GP1111 was acceptable. Therefore, the strong equivalence of PF-06438179/GP1111 to infliximab with regard to efficacy, pharmacokinetics, and safety allowed the approval of it in the treatment of IBD, which is based on the concept of extrapolation. However, data on the efficacy and safety profiles in patients with IBD are very limited. A retrospective real-life study of 87 pediatric IBD patients assessed the efficacy of several biosimilars including CT-P13, SB2 and PF-06438179/GP1111, and demonstrated their favorable effectiveness in induction and maintenance of disease remission. Another single-center observational study reported that switching from SB2 to PF-06438179/GP1111 and re-switching from PF-06438179/GP1111 to SB2 were effective and tolerated (Macaluso et al., 2023). One point should be noted is that this study only included ten patients with IBD and followed up 16–28 weeks. The small sample size and short length of follow-up may limit the strength of conclusions. Further large, multicenter, long-term, prospective studies are needed. Moreover, head-to-head parallel studies are also warranted to provide more clinical evidence and clarify the exact role in the treatment of IBD, thus building confidence in the use of PF-06438179/GP1111 in IBD.

#### 3.2.4 Others

ABP 710, a biosimilar of infliximab, was approved for CD, UC, RA, AS, psoriasis, and psoriasis arthritis by the FDA in 2019 (Generics and Biosimilars Initiative, 2023b). It presented physicochemical, pharmacodynamic, and pharmacokinetic similarities to originator infliximab based on the analytical study and phase I clinical study (Chow et al., 2020; Saleem et al., 2020). Comparable efficacy, safety, and immunogenicity profiles were also demonstrated in the comparative clinical trial of RA. The ABP 710 group showed similar ACR-20 response rates (at week 22) to the infliximab arm (68.1% vs. 59.1%). Besides, there are also no clinically meaningful differences between the two arms in AEs (51.8% vs. 49.6%) and incidence of ADAbs (57.1% vs. 60.0%) (Genovese et al., 2020). No new safety signals have been reported. Other agents including NI-071, BOW015, GB242, CMAB008, etc. have also been approved by different countries. One network meta-analysis including seven randomized controlled trials of RA demonstrated that treatment of NI-071 was more probable to gain therapeutic success (the ACR-20 response rate), compared with ABP 710, CT-P13, PF-06438179/GP1111, and SB2 (Lee and Song, 2023). BOW015, GB242, and CMAB008 were claimed to be comparable to infliximab in terms of bioavailability, safety, and immunogenicity in three phase I clinical studies (Lambert et al., 2016; An et al., 2019; Zhang et al., 2019). Non-inferiority studies of these agents were all conducted in patients with RA. The ACR-20 response rates of the GB242 group and CMAB008 at week 30 were highly similar to that of the infliximab group (62.54% vs. 56.89%, and 57.6% vs. 62.2%, respectively). No clinically meaningful differences in safety, immunogenicity, and pharmacokinetics were found (Liu et al., 2022; Ye et al., 2023). However, studies on the above agents in IBD are still in the preliminary stages, no randomized studies and real-world data were reported. Further efforts should be made to facilitate clinical equivalence study in IBD.

### 3.3 Biosimilars of adalimumab in IBD

#### 3.3.1 ABP 501

ABP 501, the first biosimilar of adalimumab, was approved for various diseases including RA, CD, UC, AS, and others by the FDA in 2016 and by the EMA in 2017(Generics and Biosimilars Initiative, 2023a; Generics and Biosimilars Initiative, 2023b). The analytical and functional characterization studies suggested that ABP 501 and adalimumab had great similarity in identity, general properties, physicochemical properties, purity and impurities, and inhibition effect on TNFα activities. The equivalent pharmacokinetics, similar safety profiles, and comparable immunogenicity of ABP 501 and adalimumab were further confirmed in a phase I study (Kaur et al., 2017). Based on the similar structures, functions, and pharmacokinetics between ABP 501 and adalimumab, further clinical equivalence studies were conducted. Comparable efficacy, safety, and immunogenicity between ABP501 and adalimumab were first demonstrated in patients with moderate to severe plaque psoriasis and then confirmed in cases with moderate to severe RA (Cohen et al., 2017; Papp et al., 2017). Following studies in IBD further claimed its favorable efficacy and safety. An observational study demonstrated that about 56% of CD patients could gain clinical remission upon ABP 501 treatment, with no new safety signals detected. Besides, the mean HBI scores (4.7 vs. 6.1) and CRP values (6.2 mg/L vs. 14.9 mg/L) at week 12 were numerically lower compared with the baseline values (Ribaldone et al., 2020). A three-arm propensity score-weighted analysis further compared the therapeutic effects and safety profiles of adalimumab and its biosimilars (ABP501 and SB5) in 86 CD and 69 UC patients. The three arms showed no significant differences in steroid-free clinical remission rates at induction stages (40.0% vs. 50.0% vs. 58.7%, at week 8) and maintenance stages (49.1% vs. 54.5% vs. 59.0%, at week 32). What should be noted is that superior efficacy was achieved by patients with CD compared with those with UC. The clinical response rate at week 8, and steroid-free clinical remission rates at weeks 8 and 32 were significantly higher in CD than in UC (Barberio et al., 2021). This is in accordance with the findings that adalimumab, infliximab, and its biosimilar were more effective in CD than UC, without no differences in safety and tolerability (Barberio et al., 2020). Underlying mechanisms are needed to be revealed.

ABP 501 seems to be as effective and tolerated as adalimumab in patients with IBD, thus providing an additional option for IBD patients who are naïve to or previously exposed to adalimumab. Switching from adalimumab to ABP 501 might be a cost-effective therapy for those patients. Available data indicated that there were no significant changes in HBI scores and CRP levels after switching from adalimumab to ABP 501 (Ribaldone et al., 2020). Similarly, Cingolani et al. (Cingolani et al., 2021) enrolled 55 IBD patients with switching treatment (adalimumab to ABP 501) and followed up for 6 months. In comparison with sustained therapy (adalimumab), switching treatment did not exert negative effects on HBI scores, PMS scores, and FC levels. There were still 76.3% of patients in remission after switching (Cingolani et al., 2021). Recently, the ADA-SWICTH study provided complementary data on disease relapse and safety profiles after switching treatment in patients with IBD (Casanova et al., 2023). Comparable relapse rates at 6 months (3% vs. 3%), 12 months (6% vs. 6%), and 24 months (26% vs. 12%) between switch treatment and sustained treatment group were recorded. The switching treatment group presented a numerally lower risk of suffering from endoscopic and/or radiologic activity compared with the other group (3% vs. 10%). Besides, this study also reported similar AEs between the two arms (6% vs. 5%), which further increased the confidence of physicians in the use of adalimumab biosimilars in clinical practice (Casanova et al., 2023). More valuable data were provided by the SPOSAB study. In this study, 85.5% of patients (adalimumab naïve) could gain clinical remission and 75.3% of patients could achieve a steroid-free remission after a 12-week ABP501 treatment. No efficacy difference was found between anti-TNFs-naïve patients and those previously exposed to anti-TNFs. However, inconsistent findings were reported by Cingolani et al. (2022). Better therapeutic effects of ABP 501 were achieved in anti-TNF-naïve patients, compared with anti-TNF-experienced ones (Cingolani et al., 2022). Different identifications of therapeutic effects in different studies may explain this inconsistency. One note in particular is that the incidence rates of SAEs were significantly lower in the switching therapy group. Thus, the lower incidence rates of SAEs might partly account for the finding that patients receiving switching therapy (adalimumab to ABP501) were more likely to persist in ABP 501 treatment, in comparison with those adalimumab-naïve patients (Macaluso et al., 2021a). Besides, no negative impacts of ABP 501 treatment on health-related quality of life were recorded, whether for the ABP 501 initiators or the adalimumab-ABP 501 switchers. More than 98% of physicians and patients expressed their satisfaction on ABP 501 treatment (Jin et al., 2024). Indeed, ABP 501 is truly effective and well-tolerated in IBD. However, data on immunogenicity, long-term efficacy, and long-term safety of ABP 501 in IBD are limited, suggesting a need to fill this gap. Cost-benefit analyses based on medical insurance of different countries are also warranted. Furthermore, in the “precision medicine” era, identifying suitable patients who will benefit most from ABP 501 is an essential prerequisite in the precision treatment of IBD. Therefore, exploring reliable biomarkers for predicting therapeutic response to ABP 501 is also needed.

#### 3.3.2 SB5

SB5 is another biosimilar of adalimumab, approved by the EMA in 2017 and the FDA in 2019(Generics and Biosimilars Initiative, 2023a; Generics and Biosimilars Initiative, 2023b). The clinical equivalence study was firstly conducted in a large phase III randomized study of 542 patients with moderate to severe RA. The ACR20, ACR50, and ACR70 response rates were equivalent between the SB5 treatment group and the adalimumab treatment group. No significant differences in the incidence of TEAEs, development of ADAbs, and pharmacokinetics were reported in this study (Weinblatt et al., 2018). By extrapolation, the approval was extended to IBD, axial spondylarthritis, and psoriasis arthritis (Müller-Ladner et al., 2023).

Lukas et al. (2020) firstly provided the real-life study that directly compared the efficacy, safety, pharmacokinetic, and immunogenicity profiles between the originator and SB5. 93 IBD patients received switch treatment (from adalimumab to SB5) and the other 93 patients still received originator adalimumab therapy. The two groups did not show any significant changes at week 10 with regard to HBI scores, PMS scores, CRP levels, and FC concentrations. They also claimed no notable differences in trough drug concentrations (13.0 μg/mL vs. 13.7 μg/mL) and the incidence of ADAbs (2% vs. 2%) between the two arms at week 10. However, the follow-up time was only 10 weeks, too short to evaluate the long-term safety profiles. Further study conducted by Barberio et al. (2021) provided additional information on the long-term efficacy and safety profiles in patients with IBD. They compared the effectiveness and safety profiles of SB5 and adalimumab at weeks 8 and 48. The rates of steroid-free clinical remission at the two time points were 58.7% and 59.0%, which were comparable to the rates of adalimumab (40% and 49.1%, respectively). Similar clinical response rates at weeks 8 and 48 were also collected in this study (Barberio et al., 2021). Data on the 1-year performance of SB5 in patients with IBD were further reported by a UK study. They divided patients into two arms, the SB5-switch group and the SB5-start group (adalimumab naïve), and followed up at a median time of 13.7 months and 8.3 months, respectively. SB5 showed comparable effectiveness to adalimumab given similar 1-year drug persistence rates (62.5% vs. 50.89%) (Chen et al., 2019; Derikx et al., 2021). Switching treatment also did not worsen the biochemical remission rates, fecal biomarker remission rates, and clinical remission rates at weeks 26 and 52. Besides, there were also no differences in the median C_trough_ concentrations at weeks 0, 26, and 52 after switching treatment (10.1 μg/mL vs. 11.6 μg/mL vs. 7.8 μg/mL). This is consistent with the other two studies reporting stable trough drug concentrations after switching from adalimumab to SB5 (Lukas et al., 2020; Tapete et al., 2022).

About 19.9% of patients in the SB5-switch cohort and 17.3% of patients in the SB5-start cohort reported AEs, respectively. The most common AE in the SB5-switch cohort was injection-site pain (66.7%), which lead to a double-switch treatment (from adalimumab to SB5 to ABP 501) in these patients. Therefore, this study provided the first data on the double-switch treatment. Median trough concentrations were stable during the first and the second switch treatment, suggesting that multiple switches might work in cases intolerant to SB5 (Derikx et al., 2021). Similarly, switching from adalimumab to ABP 501 to SB5 was also tolerated (Ribaldone et al., 2021). It did not impair the efficacy and increase the risk of AEs in patients with IBD. Given that injection-site pain negatively affected treatment persistence, solutions to help relieve injection-site pain were designed. A citrate-free and high concentration of SB5 (SB5-HC) was claimed to be associated with less injection site pain (Ahn et al., 2022). Besides, injection technique training and psychological interventions are also important to alleviate pain. Overall, SB5 is effective and safe in IBD, though this conclusion was based on post-marketing evidence. Healthcare professionals and patients expressed their concerns about its efficacy and safety, causing some negative effects on the market share of SB5. Therefore, further randomized controlled clinical trials may help build confidence and increase the uptake of SB5. Moreover, some studies did not perform dose optimization in a standardized manner and evaluate endoscopic/histological healing after dose optimization, which might cause potential selection bias.

#### 3.3.3 BI 695501

BI 695501 is another biosimilar of adalimumab (Wynne et al., 2016). The regulatory approval of BI 695501 was granted in Europe and the United States in 2017 based on the “totality of the evidence” (Generics and Biosimilars Initiative, 2023a; Generics and Biosimilars Initiative, 2023b). The bioequivalence, comparable safety, and similar immunogenicity of BI 695501 to adalimumab were first demonstrated in a phase I study of 327 healthy volunteers in 2016 (Wynne et al., 2016). Two years later, the efficacy data were primarily obtained in patients with RA (Cohen et al., 2018a). This study suggested the non-inferiority of BI 695501 to originator adalimumab in terms of efficacy, safety, and immunogenicity. Switching from adalimumab to BI 695501 was not associated with lower efficacy, increased incidence of AEs, and elevated levels of ADAbs (Cohen et al., 2018a). By extrapolation, the approval was extended to other indications including CD, UC, AS, psoriasis, and others.

Clinical data on BI 695501 in patients with IBD were limited. One large, multicenter, randomized, double-blind, phase 3 study including 147 moderately to severely active CD patients divided patients into two groups, the BI 695501 group and the adalimumab group (Hanauer et al., 2021). The two groups showed similarities in clinical response rates (90% vs. 94% at week 4, and 81% vs. 82% at week 24), clinical remission rates (68% vs. 75% at week 24), and AE rates (63% vs. 56% at week 24). Besides, this VOLTAIRE-CD study also evaluated the feasibility of switching from adalimumab to BI 695501. No negative impacts of switching treatment on efficacy and AEs were claimed. Patients in the switch group presented a similar degree of reduction in CDAI scores and a similar incidence of TEAEs to those in the BI 695501 sustained group (Hanauer et al., 2021). Likewise, the VOLTAIRE-X study of 238 patients with chronic plaque psoriasis further demonstrated that switching back and forth from adalimumab to BI 695501 was safe, effective, and tolerated (Menter et al., 2022). This study provided direct evidence for the interchangeability of BI 695501. Thus, BI 695501 (Cyltezo) became the first FDA-approved interchangeable biosimilar to adalimumab (Kay et al., 2024). This indicated that pharmacists can substitute the biosimilar for its originator without the permission of the prescribing healthcare professionals (Alvarez et al., 2020). The “interchangeable” logo may greatly increase the uptake of Cyltezo. More treatment options are thus provided for patients who need repeated therapy during the overall disease course. However, the paucity of safety and immunogenicity data on IBD highlighted the need to conduct real-world studies in the future. Besides, evaluating the long-term outcomes of BI 695501 on the basis of interchangeability designation in different diseases is also warranted.

#### 3.3.4 GP2017

GP2017 is the fourth adalimumab biosimilar approved by the EMA and the third one approved by the FDA in 2018 (Generics and Biosimilars Initiative, 2023a; Generics and Biosimilars Initiative, 2023b). The equivalent efficacy, safety, and immunogenicity between GP2017 and adalimumab were demonstrated in a phase III randomized study of 465 patients with plaque psoriasis. This study also demonstrated that multiple switches between adalimumab and GP2017 did not impair the disease outcomes and affect the safety and immunogenicity profiles (Blauvelt et al., 2018). The following study in patients with moderate to severe active RA further confirmed the non-inferiority of GP2017 to adalimumab in terms of efficacy, safety, and immunogenicity (Wiland et al., 2020). GP2017 was approved for IBD through extrapolation of indications.

Real-life data on the efficacy of GP2017 in IBD were provided by an Italy study (Mocci et al., 2022). This study retrospectively analyzed the clinical data of 134 patients with IBD. Among these patients, 62 patients received GP2017 treatment while the others received adalimumab therapy. Similar clinical remission rates and clinical response rates were reported regardless of whether they were naïve to biologics or not. 82.3% of patients in the GP2017 group and 75.0% of patients in the adalimumab group achieved clinical remission at a median follow-up time of 12 months. No clinically meaningful differences in the rates of treatment optimization and surgery, as well as the incidence of AEs were suggested. More importantly, GP2017 showed better effects in achieving mucosal healing than adalimumab. The mucosal healing rate in the GP2017 group was about 1.5 times as that in the adalimumab group (89.2% vs. 60.2%). Recently, another real-world retrospective study evaluated the impacts of switching treatment in IBD patients (Vernero et al., 2023). Switching from adalimumab to GP2017 did not increase the clinical disease activity and interfere the treatment persistence. Patients who were previously exposed to infliximab were at a higher risk of needing dose optimization of GP 2017 (Vernero et al., 2023). However, the retrospective design cannot prove the causal association and control the potential confounding factors. Well-designed, well-paired, prospective studies might add useful information. A prospective observational study of 50 IBD patients further proved the favorable efficacy and safety profiles of GP2017. 75.0% of patients obtained remission or partial response after 12-week treatment of GP2017. A median decrease of CDAI and Mayo score was 140.5 and 4.0. respectively (Wasserbauer et al., 2022). This study also had limitations, including a lack of reference product control, a short follow-up time, and a small sample size. Recently, a cross-sectional, questionnaire-based study assessed the subjective efficacy of switching treatment in 179 IBD patients (Sarlós et al., 2023). Patients with GP2017 switching treatment reported better efficacy of GP2017 than adalimumab. However, they also complained of a higher incidence of new AEs (1.79 per patient) that did not occur during adalimumab treatment. Most of these patients also expressed their willingness to switch back to adalimumab if possible (Sarlós et al., 2023). Such a contradiction may be partly explained by the “nocebo” effect, an unfavorable therapeutic effect of a medical therapy that is not caused by pharmacological effects and is related to patients’ high expectations on it (Colloca et al., 2019).

Overall, GP2017 is as effective and safe as adalimumab in patients with IBD. However, there is relatively limited data on the pharmacokinetics and immunogenicity of GP2017 in IBD. Little is known about the drug concentrations and ADAb levels after switching treatment. More prospective studies are also needed to evaluate the performance of multiple switches between adalimumab and GP2017 in patients with IBD.

#### 3.3.5 Others

Biosimilars of adalimumab including FKB327, MSB11022, AVT02, PF-06410293, CHS-1420, CT-P17, and others were also approved for treatment of CD and UC (Generics and Biosimilars Initiative, 2023b; Generics and Biosimilars Initiative, 2023a). However, most clinical evidence was obtained from patients with RA and plaque psoriasis. There were relatively limited efficacy and safety data on them in IBD. FKB327 treatment showed high efficacy in inducing and maintaining disease remission or partial response at week 12 (18/22, 81.8%), which was comparable to the effectiveness of GP2017 (21/27, 75.0%) (Wasserbauer et al., 2022). A large, multicenter, observational study of 533 IBD patients evaluated the efficacy and safety profiles of four biosimilars of adalimumab (SB5, APB501, GP2017, and MSB11022). Available data indicated that 81.8% of patients with MSB11022 could achieve clinical remission, similar to SB5 (75.2%), APB501 (78.3%), and GP2017 (77.5%). MSB11022 also showed similarities in steroid-free remission rates and mucosal healing rates to the other three biosimilars. No new safety concerns were identified in MSB11022 (Tursi et al., 2023). However, the data must be viewed critically because the patients included in the MSB11022 group were only 11, which may weaken the strength of the evidence. Another Italy study of 143 IBD patients further compared the efficacy and safety of the four biosimilars (SB5, APB501, GP2017, and MSB11022) after switching from adalimumab. No significant differences in remission maintenance rates between the four biosimilars were claimed (Tursi et al., 2022). However, the sample size of the MSB11022 group was still too small (3 patients), which suggested a need to conduct a larger study of MSB11022. There are few studies on the roles of AVT02, PF-06410293, CHS-1420, and CT-P17 in patients with IBD. One phase IV clinical trial (NCT05913817) is currently evaluating the effectiveness, safety, and tolerability of AVT02 in patients who switch from low-concentration adalimumab to AVT02 (Clinical Trials.gov, 2024). CD and UC patients are included in this study. Most studies focused on healthy subjects and plaque psoriasis patients. More real-world studies in patients with IBD are therefore needed.

## 4 The benefits of biosimilars

Based on extrapolation, biosimilars of anti-TNFα were approved for a variety of immune-mediated diseases including CD and UC. Biosimilars showed strong bioequivalence and similar efficacy results, as well as comparable safety and immunogenicity profiles to originators. In the absence of high-quality evidence from randomized controlled trials, healthcare professionals always relied on real-life data to support their use in clinical settings. Even so, many physicians and patients still choose biosimilars, based on the following reasons.

### 4.1 Cost-saving

The most important benefit of using biosimilars is the cost savings. Available data suggested that biologics accounted for 77% of prescription drug spending in 2017 and made up 92% of spending growth from 2006 to 2017 under Medicare Part B (Dickson and Kent, 2021). According to the U.S. Generic and Biosimilar Medicines Savings Report 2023 provided by the Association for Accessible Medicines, the cumulative cost savings of biosimilars from 2015 to 2022 in the United States were $23.6 billion, which will increase to $130 billion in 2025. What should be noted is that biosimilars of infliximab accounted for the most of savings ($3.3 billion), indicating a pivotal role of infliximab biosimilars in cost savings (Association for Accessible Medicines, 2023).

It is universally acknowledged that the introduction of biosimilars greatly decreased medical spending. Take infliximab biosimilars for example, both the list price and net price of infliximab increased at a rate of 6% from 2007 to 2013. The introduction of its biosimilars decreased the net price to a mean of −13.6% in 2019 (San-Juan-Rodriguez et al., 2019). Furthermore, the cost savings resulting from the introduction of infliximab biosimilars in the United States were $21 million from 2015 to 2019 under Medicare Part B (Dickson and Kent, 2021). As for adalimumab originator (Humira), the list price increased from 2013 to 2020 continuously ($2,784 in 2020 vs. $1,153 in 2013), which caused a huge burden on public and private payers. However, the 2023 list price for the biosimilar of adalimumab (Amjevita) was only $1,558, a 44% discount from the 2020 list price of Humira (Dickson et al., 2023). This may hold promise for slowing prescription drug spending growth to some extent. In Europe, the cumulative cost savings of Remsima in 2014 were €25.79∼€77.37 million over a 1-year time horizon in Germany, the United Kingdom, Italy, the Netherlands, and Belgium (Jha et al., 2015). In the United Kingdom, Italy, France, and Germany, using of CT-P13 over 5 years brought greater savings (€233∼€433.5 million) for RA patients and payers. It was estimated that the potential cost savings were enough to cover biosimilar treatment for another 7,500 more patients with RA (Dörner et al., 2016). What should be noted is that changing prices of originators and biosimilars made it very challenging to do a real cost benefit evaluation of biosimilars. Researchers should do financial analysis based on the actual status. A recent report demonstrated that the median biosimilar treatment costs per patient-month in the United States in 2020 were $8,987, lower than originators ($11,503). Similar findings were also reported in Germany ($932 vs. $1,285) and Switzerland ($1,351 vs. $1,801) (Carl et al., 2022). Biosimilars have relatively lower prices than originators. Price negotiation and demand-side measures were carried out to facilitate market entry and market share. As a result, the entry of biosimilars further drove stiff competition between pharmaceutical companies. Manufacturers then reduced the price of originators to gain market share. Based on data from 2020, the introduction of the first and the second biosimilars of infliximab markedly decreased the volume-weighted average price per defined daily dose by 13.6% and 26.4% in Europe, respectively (Car et al., 2023). In the United States, market entry of biosimilars of infliximab decreased the average sales price of originators by 58%. Based on data from 2022, biosimilars were claimed to reduce the growth rate of total autoimmune disease spending by 41% (Association for Accessible Medicines, 2023). Collectively, biosimilars hold promise for curbing the prescription drug spending growth and lowering government expenditures.

### 4.2 Increase patient access to biologics

The high price of originator biologics substantially limited patient access to them. Many patients, especially those low-income patients, cannot afford the high costs of biologics. Biosimilars showed their superiority in prices and thus attracted more attention from patients. Besides, government and healthcare managers proposed relevant policies to promote biosimilar use and increase their uptake. In Europe, market entry of biosimilars significantly increased the utilization of infliximab and adalimumab by an average of 88.9% and 22.4%, respectively (Car et al., 2023). Biosimilars have been used in 5.8 billion days of patient therapy over the last 10 years in Europe, increasing patient treatment days significantly (IQVIA Institute, 2023a). According to the U.S. Generic and Biosimilar Medicines Savings Report 2023, the cumulative patient treatment days were 694 million days since 2015, which made more than 344 million incremental days of patient therapy (Association for Accessible Medicines, 2023). These data indicated that biosimilars expand access to biologic treatment and healthcare. As it is known to us, inadequate or inappropriate treatment may aggravate disease progression, especially in IBD patients with severe disease (Zeng et al., 2023). Biosimilars provide an additional option for these patients, making it possible for patients to receive biologic treatment earlier and receive dose optimization more easily. As a result, the disease prognosis might be improved and natural history might be changed.

## 5 The challenges and obstacles of biosimilars

Although biosimilars are cost-saving, the market share of biosimilars varies across different countries (IQVIA Institute, 2023b). Available data suggested that the uptake for biosimilars of infliximab was lowest in the United States, with the highest uptake for bevacizumab biosimilars (36% vs. 3%) in 1 year after their entry into the market. In general, Germany has the highest market share of biosimilars, followed by the United States and Switzerland (Carl et al., 2022). However, the adoption of infliximab biosimilars increased to 44% 6 years after market entry in the United States (IQVIA Institute, 2023a). This difference in the market share of biosimilars might partly explained by different policies for market entry, reimbursement, and drug pricing negotiation across different countries.

The extensive patent protections and complex patent litigation on originators became the major threat to market entry of biosimilars. Even though biosimilars can get approval, patent infringement damages discourage them from entering the market. Take Humira for example, AbbVie company registered more than 160 patents on Humira which do not expire until 2037, though the core compound patent expired 7 years ago (Kvien et al., 2022). The tough situation made biosimilar companies have to sign settlement agreements and make major concessions. Otherwise, huge compensation and legal costs might be paid.

As for reimbursement, take the United States for example, drugs with lower average sales prices bring a lower reimbursement for insurers, pharmacy benefit managers (PBMs), government, wholesalers, and retailers. As a result, they prefer to choose high-priced originators, in order to receive higher rebates, thus hindering market penetration of low-priced biosimilars. Besides, some manufacturers proposed unique contracting mechanisms including rebate traps. It means that insurers, PBMs, and clinicians should return the rebates they got from prescribing originators if patients start to use biosimilars (Dean et al., 2021). Moreover, lack of and/or delayed coverage further delayed the adoption rates of biosimilars. Medicare, Medicaid, and commercial insurance are unwilling to cover the costs of biosimilars due to the great rebates offered by originator manufacturers. In 2023, Medicare price negotiation was launched in the United States. Although biosimilars were not included in the list, it will make an impact on biosimilars to some extent.

Interchangeability is another obstacle to biosimilars. Due to the rigorous standards set in the United States, the number of interchangeable biosimilars was relatively small. Pharmacists cannot substitute the biosimilar for its originator automatically, further resulting in a lower market share of biosimilars. What should be noted is that interchangeability is not permitted in many other countries, suggesting a need to analyze biosimilar issues based on national conditions.

In China, the coverage of commercial insurance is significantly lower than in other developed countries (Xu et al., 2021). Some biosimilars were not only not covered by basic medical insurance, but also not covered by commercial insurance, which further decreased the accessibility and affordability of biosimilars. Besides, the drug price negotiation mechanism of China is also different from other countries. The United States carried out independent pricing. Manufacturers, insurers, and PBMs fix the price by pricing negotiation. While in China, the drug price is based on manufacturing costs and clinical values. The National Healthcare Security Administration directly negotiated with manufacturers and fixed prices. Most manufacturers want to increase their market share at the expense of decreased drug prices. However, the biosimilar market in China is frail. And sales of biosimilars are not satisfactory, which always leads to failure in biosimilar pricing negotiation. A vicious circle developed. Therefore, more incentive programs are needed in China.

In addition to the above policies, the prescription inertia of physicians and low patient acceptance are also important obstacles to increasing market share. Healthcare professionals are willing to prescribe brand drugs that are used frequently, given that they are good at using them and dealing with side reactions caused by these drugs. Besides, the efficacy and safety of biosimilars are still a major concern, although they were proven to have comparable efficacy and safety to originators. Moreover, more concerns about therapeutic responses and side reactions were raised when making switching treatments, especially in patients in disease remission.

The above challenges and obstacles indeed hinder the development of biosimilars. A collaboration between government agencies, state legislators, manufacturers, insurance companies, healthcare professionals, and patients is encouraged.

## 6 The future of biosimilars in IBD

Biosimilars do play a key role in the treatment of IBD. With the expiration of patents of some anti-TNFα biologics, an increasing number of anti-TNFα biosimilars entered the market. The equivalent efficacy, safety, and immunogenicity profiles between biosimilars and originators were validated in several clinical trials. However, most clinical trials were not conducted in patients with IBD, resulting in some concerns about the efficacy and safety in IBD. This further discouraged the market share of biosimilars in IBD treatment, suggesting a pressing need to design more studies to confirm their roles in IBD patients. With an increasing number of alternative biosimilars for IBD patients, more efforts should also be put into the investigation of efficacy and immunogenicity profiles of multiple successive switches between originators and biosimilars. Besides, from the perspectives of pharmacoeconomics and health economics, cost-effectiveness analyses of biosimilars are also warranted.

In the era of precision medicine, precision diagnosis and precision treatment hold the key to disease management. Given that IBD is a progressive disease, tailoring an individualized and precise therapeutic plan within the window of opportunity is highly crucial. Combined analysis of clinical, genetic, epigenetic, serological, histological, and fecal markers may assist physicians in predicting therapeutic response and selecting a suitable drug for individuals (Chen P. et al., 2020; Chen et al., 2023; Yueying et al., 2023). Thus, exploring predictive markers and establishing predictive models of anti-TNFα biosimilars seems to be necessary. Identifying molecular markers for therapeutic drug monitoring will also be helpful in the precision monitoring of biosimilars. To achieve the therapeutic goal for IBD, “treat-to-target,” determining the optimal switching time for IBD patients is also of great importance. Switching too early may cause disease flare, while switching too late may increase the medical costs of patients. Moreover, more importance should be attached to ADAbs and drug concentrations. ADAbs are closely correlated with adverse reactions and therapeutic failures. Available assay techniques for ADAb detection and drug concentration assessment include enzyme-linked immunosorbent assay, fluid phase radioimmunoassay, homogeneous mobility shift assay, and others (Soubières and Poullis, 2016; Strand et al., 2020). The sensitivity and specificity of them vary greatly. The lack of a gold standard assay, undefined threshold values, and undetermined detection time points make it difficult to interpret immunogenicity results. Therefore, it is definitely a pressing need to identify a gold standard assay, and a universally acknowledged threshold value and detection time point for biosimilar treatment. What’s more, the challenge remains to differentiate ADAbs from biologics themselves or other endogenous antibodies, which further limits their clinical application (Strand et al., 2020). More importantly, designing biosimilars with comfortable routes of administration (such as subcutaneous administration), high concentration, and reduced immunogenicity also became a matter of prime importance.

The European Union (EU) and the United States have established a comparatively perfect regulatory framework for biosimilar discovery, approval, and supervision. However, the study on anti-TNFα biosimilars in China is in the preliminary stages. Relevant laws and regulations are not very sound. Dynamically assessing and revising cost-containment and use restriction policies is the essential prerequisite for ensuring a competitive and sustainable market for biosimilar competition. More efforts are also needed to accelerate the discovery and approval processes of biosimilars. Patient empowerment and medication self-management will be the future medical model. Thus, education and training must be provided to build their confidence in using biosimilars. A close collaboration between government agencies, state legislators, manufacturers, insurance companies, healthcare professionals, and IBD patients is also needed, which holds the key to boosting the development of biosimilars.

## References

[B1] Abraham C. Cho J. H. (2009). Inflammatory bowel disease. N. Engl. J. Med. 361 (21), 2066–2078. 10.1056/NEJMra0804647 19923578 PMC3491806

[B2] Ahn S. S. Lee M. Baek Y. Lee S. (2022). A randomized pharmacokinetic study in healthy male subjects comparing a high-concentration, citrate-free SB5 formulation (40 mg/0.4 ml) and prior SB5 (adalimumab biosimilar). Rheumatol. Ther. 9 (4), 1157–1169. 10.1007/s40744-022-00471-8 35776269 PMC9309445

[B3] Alvarez D. F. Wolbink G. Cronenberger C. Orazem J. Kay J. (2020). Interchangeability of biosimilars: what level of clinical evidence is needed to support the interchangeability designation in the United States? BioDrugs 34 (6), 723–732. 10.1007/s40259-020-00446-7 32990892 PMC7669758

[B4] An Q. Zheng Y. Zhao Y. Liu T. Guo H. Zhang D. (2019). Physicochemical characterization and phase I study of CMAB008, an infliximab biosimilar produced by a different expression system. Drug Des. Devel Ther. 13, 791–805. 10.2147/dddt.S170913 PMC6420106 30880912

[B5] Association for Accessible Medicines (2023). The U.S. Generic and biosimilar medicines savings report 2023. Available at: https://accessiblemeds.org/sites/default/files/2023-09/AAM-2023-Generic-Biosimilar-Medicines-Savings-Report-web.pdf (Accessed March 30, 2024).

[B6] Barberio B. Cingolani L. Canova C. Barbieri G. Sablich R. Urbano M. T. (2021). A propensity score-weighted comparison between adalimumab originator and its biosimilars, ABP501 and SB5, in inflammatory bowel disease: a multicenter Italian study. Ther. Adv. Gastroenterol. 14, 17562848211031420. 10.1177/17562848211031420 PMC8295962 34349836

[B7] Barberio B. Gracie D. J. Black C. J. Ford A. C. (2023). Efficacy of biological therapies and small molecules in induction and maintenance of remission in luminal Crohn's disease: systematic review and network meta-analysis. Gut 72 (2), 264–274. 10.1136/gutjnl-2022-328052 35907636

[B8] Barberio B. Zingone F. D'Incà R. Rovigo L. Bertani L. Bodini G. (2020). Infliximab originator, infliximab biosimilar, and adalimumab are more effective in crohn's disease than ulcerative colitis: a real-life cohort study. Clin. Transl. Gastroenterol. 11 (5), e00177. 10.14309/ctg.0000000000000177 32677808 PMC7263644

[B9] Ben-Horin S. Vande Casteele N. Schreiber S. Lakatos P. L. (2016). Biosimilars in inflammatory bowel disease: facts and fears of extrapolation. Clin. Gastroenterol. Hepatol. 14 (12), 1685–1696. 10.1016/j.cgh.2016.05.023 27215364

[B10] Bittner B. Richter W. Schmidt J. (2018). Subcutaneous administration of biotherapeutics: an overview of current challenges and opportunities. BioDrugs 32 (5), 425–440. 10.1007/s40259-018-0295-0 30043229 PMC6182494

[B11] Blandizzi C. Meroni P. L. Lapadula G. (2017). Comparing originator biologics and biosimilars: a review of the relevant issues. Clin. Ther. 39 (5), 1026–1039. 10.1016/j.clinthera.2017.03.014 28416374

[B12] Blauvelt A. Lacour J. P. Fowler J. F. Jr. Weinberg J. M. Gospodinov D. Schuck E. (2018). Phase III randomized study of the proposed adalimumab biosimilar GP2017 in psoriasis: impact of multiple switches. Br. J. Dermatol 179 (3), 623–631. 10.1111/bjd.16890 29917226

[B13] Bouhnik Y. Carbonnel F. Laharie D. Stefanescu C. Hébuterne X. Abitbol V. (2018). Efficacy of adalimumab in patients with Crohn's disease and symptomatic small bowel stricture: a multicentre, prospective, observational cohort (CREOLE) study. Gut 67 (1), 53–60. 10.1136/gutjnl-2016-312581 28119352 PMC5754855

[B14] Bouhnik Y. Fautrel B. Beaugerie L. Pelletier A. L. Martinez-Vinson C. Freudensprung U. (2023). PERFUSE: a French non-interventional study of patients with inflammatory bowel disease receiving infliximab biosimilar SB2: a 12-month analysis. Ther. Adv. Gastroenterol. 16, 17562848221145654. 10.1177/17562848221145654 PMC10021102 36936799

[B15] Buchner A. M. Schneider Y. Lichtenstein G. R. (2021). Biosimilars in inflammatory bowel disease. Am. J. Gastroenterol. 116 (1), 45–56. 10.14309/ajg.0000000000000844 33110013

[B16] Burisch J. Vardi H. Schwartz D. Friger M. Kiudelis G. Kupčinskas J. (2020). Health-care costs of inflammatory bowel disease in a pan-European, community-based, inception cohort during 5 years of follow-up: a population-based study. Lancet Gastroenterol. Hepatol. 5 (5), 454–464. 10.1016/s2468-1253(20)30012-1 32061322

[B17] Car E. Vulto A. G. Houdenhoven M. V. Huys I. Simoens S. (2023). Biosimilar competition in European markets of TNF-alpha inhibitors: a comparative analysis of pricing, market share and utilization trends. Front. Pharmacol. 14, 1151764. 10.3389/fphar.2023.1151764 37153785 PMC10160635

[B18] Carl D. L. Laube Y. Serra-Burriel M. Naci H. Ludwig W. D. Vokinger K. N. (2022). Comparison of uptake and prices of biosimilars in the US, Germany, and Switzerland. JAMA Netw. Open 5 (12), e2244670. 10.1001/jamanetworkopen.2022.44670 36459139 PMC9719051

[B19] Casanova M. J. Nantes Ó. Varela P. Vela-González M. Rivero M. Sierra-Gabarda O. (2023). Real-world outcomes of switching from adalimumab originator to adalimumab biosimilar in patients with inflammatory bowel disease: the ADA-SWITCH study. Aliment. Pharmacol. Ther. 58 (1), 60–70. 10.1111/apt.17525 37089065

[B20] Chaparro M. Garre A. Guerra Veloz M. F. Vázquez Morón J. M. De Castro M. L. Leo E. (2019). Effectiveness and safety of the switch from Remicade® to CT-P13 in patients with inflammatory bowel disease. J. Crohns Colitis 13 (11), 1380–1386. 10.1093/ecco-jcc/jjz070 30976785

[B21] Chen C. Hartzema A. G. Xiao H. Wei Y. J. Chaudhry N. Ewelukwa O. (2019). Real-world pattern of biologic use in patients with inflammatory bowel disease: treatment persistence, switching, and importance of concurrent immunosuppressive therapy. Inflamm. Bowel Dis. 25 (8), 1417–1427. 10.1093/ibd/izz001 30839057

[B22] Chen H. Wu X. Xu C. Lin J. Liu Z. (2021). Dichotomous roles of neutrophils in modulating pathogenic and repair processes of inflammatory bowel diseases. Precis. Clin. Med. 4 (4), 246–257. 10.1093/pcmedi/pbab025 35692862 PMC8982532

[B23] Chen L. Li J. Ye Z. Sun B. Wang L. Chen Y. (2020a). Anti-high mobility group box 1 neutralizing-antibody ameliorates dextran sodium sulfate colitis in mice. Front. Immunol. 11, 585094. 10.3389/fimmu.2020.585094 33193406 PMC7661783

[B24] Chen L. Wang Y. Zhou H. Liang Y. Zhu F. Zhou G. (2024). The new insights of hyperbaric oxygen therapy: focus on inflammatory bowel disease. Precis. Clin. Med. 7 (1), pbae001. 10.1093/pcmedi/pbae001 38344218 PMC10858389

[B25] Chen P. Zhou G. Lin J. Li L. Zeng Z. Chen M. (2020b). Serum biomarkers for inflammatory bowel disease. Front. Med. (Lausanne) 7, 123. 10.3389/fmed.2020.00123 32391365 PMC7188783

[B26] Chen R. Li L. Tie Y. Chen M. Zhang S. (2023). Trajectory of fecal lactoferrin for predicting prognosis in ulcerative colitis. Precis. Clin. Med. 6 (3), pbad022. 10.1093/pcmedi/pbad022 38025971 PMC10680133

[B27] Choe J. Y. Prodanovic N. Niebrzydowski J. Staykov I. Dokoupilova E. Baranauskaite A. (2017). A randomised, double-blind, phase III study comparing SB2, an infliximab biosimilar, to the infliximab reference product Remicade in patients with moderate to severe rheumatoid arthritis despite methotrexate therapy. Ann. Rheum. Dis. 76 (1), 58–64. 10.1136/annrheumdis-2015-207764 26318384 PMC5264229

[B28] Chow V. Oh M. Gessner M. A. Fanjiang G. (2020). Pharmacokinetic similarity of ABP 710, a proposed biosimilar to infliximab: results from a randomized, single-blind, single-dose, parallel-group study in healthy subjects. Clin. Pharmacol. Drug Dev. 9 (2), 246–255. 10.1002/cpdd.738 31628783 PMC7027815

[B29] Cingolani A. Felice C. Lombardi G. Onidi F. M. Checchin D. Colucci R. (2022). P398 Long term efficacy and safety of ABP, 501 adalimumab biosimilar in inflammatory bowel diseases: preliminary results from the ADASWITCH study. J. Crohn's Colitis 16 (Suppl. ment_1), i392–i393. 10.1093/ecco-jcc/jjab232.525

[B30] Cingolani L. Barberio B. Zingone F. Ferronato A. Bertani L. Costa F. (2021). Adalimumab biosimilars, ABP501 and SB5, are equally effective and safe as adalimumab originator. Sci. Rep. 11 (1), 10368. 10.1038/s41598-021-89790-4 33990652 PMC8121777

[B31] Clinical Trials.gov (2024). The evaluation of injection site pain and adherence in patients switching from a low to high concentration adalimumab (AVT-02) across multiple indications. (EASE PAIN). Available at: https://clinicaltrials.gov/study/NCT05913817?limit=25&term=NCT05913817&rank=1 (Accessed March 30, 2024).

[B32] Cohen S. Genovese M. C. Choy E. Perez-Ruiz F. Matsumoto A. Pavelka K. (2017). Efficacy and safety of the biosimilar ABP 501 compared with adalimumab in patients with moderate to severe rheumatoid arthritis: a randomised, double-blind, phase III equivalence study. Ann. Rheum. Dis. 76 (10), 1679–1687. 10.1136/annrheumdis-2016-210459 28584187 PMC5629940

[B33] Cohen S. B. Alonso-Ruiz A. Klimiuk P. A. Lee E. C. Peter N. Sonderegger I. (2018a). Similar efficacy, safety and immunogenicity of adalimumab biosimilar BI 695501 and Humira reference product in patients with moderately to severely active rheumatoid arthritis: results from the phase III randomised VOLTAIRE-RA equivalence study. Ann. Rheum. Dis. 77 (6), 914–921. 10.1136/annrheumdis-2017-212245 29514803 PMC5965346

[B34] Cohen S. B. Alten R. Kameda H. Hala T. Radominski S. C. Rehman M. I. (2018b). A randomized controlled trial comparing PF-06438179/GP1111 (an infliximab biosimilar) and infliximab reference product for treatment of moderate to severe active rheumatoid arthritis despite methotrexate therapy. Arthritis Res. Ther. 20 (1), 155. 10.1186/s13075-018-1646-4 30053896 PMC6063022

[B35] Colloca L. Panaccione R. Murphy T. K. (2019). The clinical implications of nocebo effects for biosimilar therapy. Front. Pharmacol. 10, 1372. 10.3389/fphar.2019.01372 31849647 PMC6895996

[B36] Colombel J. F. Sandborn W. J. Rutgeerts P. Enns R. Hanauer S. B. Panaccione R. (2007). Adalimumab for maintenance of clinical response and remission in patients with Crohn's disease: the CHARM trial. Gastroenterology 132 (1), 52–65. 10.1053/j.gastro.2006.11.041 17241859

[B37] Dang Y. Ma C. Chen K. Chen Y. Jiang M. Hu K. (2023). The effects of a high-fat diet on inflammatory bowel disease. biomolecules 13 (6), 905. 10.3390/biom13060905 37371485 PMC10296751

[B38] Dean E. B. Johnson P. Bond A. M. (2021). Physician, practice, and patient characteristics associated with biosimilar use in Medicare recipients. JAMA Netw. Open 4 (1), e2034776. 10.1001/jamanetworkopen.2020.34776 33502485 PMC7841457

[B39] Derikx L. Dolby H. W. Plevris N. Lucaciu L. Rees C. S. Lyons M. (2021). Effectiveness and safety of adalimumab biosimilar SB5 in inflammatory bowel disease: outcomes in originator to SB5 switch, double biosimilar switch and bio-naïve SB5 observational cohorts. J. Crohns Colitis 15 (12), 2011–2021. 10.1093/ecco-jcc/jjab100 34089587 PMC8684477

[B40] Dickson S. R. Gabriel N. Hernandez I. (2023). Contextualizing the price of biosimilar adalimumab based on historical rebates for the original formulation of branded adalimumab. JAMA Netw. Open 6 (7), e2323398. 10.1001/jamanetworkopen.2023.23398 37440233 PMC10346129

[B41] Dickson S. R. Kent T. (2021). Association of generic competition with price decreases in physician-administered drugs and estimated price decreases for biosimilar competition. JAMA Netw. Open 4 (11), e2133451. 10.1001/jamanetworkopen.2021.33451 34779844 PMC8593762

[B42] Dörner T. Strand V. Cornes P. Gonçalves J. Gulácsi L. Kay J. (2016). The changing landscape of biosimilars in rheumatology. Ann. Rheum. Dis. 75 (6), 974–982. 10.1136/annrheumdis-2016-209166 26964144 PMC4893105

[B43] Fiorino G. Danese S. (2014). The biosimilar road in inflammatory bowel disease: the right way? Best. Pract. Res. Clin. Gastroenterol. 28 (3), 465–471. 10.1016/j.bpg.2014.04.006 24913385

[B44] Fiorino G. Manetti N. Armuzzi A. Orlando A. Variola A. Bonovas S. (2017). The PROSIT-BIO cohort: a prospective observational study of patients with inflammatory bowel disease treated with infliximab biosimilar. Inflamm. Bowel Dis. 23 (2), 233–243. 10.1097/mib.0000000000000995 28092307

[B45] Fischer S. Cohnen S. Klenske E. Schmitt H. Vitali F. Hirschmann S. (2021). Long-term effectiveness, safety and immunogenicity of the biosimilar SB2 in inflammatory bowel disease patients after switching from originator infliximab. Ther. Adv. Gastroenterol. 14, 1756284820982802. 10.1177/1756284820982802 PMC7812413 33505519

[B46] Food and Drug Administration (2021). Highlights of prescribing information of infliximab. Available at: https://www.accessdata.fda.gov/drugsatfda_docs/label/2021/103772s5401lbl.pdf (Accessed July 11, 2024).

[B47] Fuhr U. Tuculanu D. Berghout A. Balser S. Schwebig A. Saenger P. (2010). Bioequivalence between novel ready-to-use liquid formulations of the recombinant human GH Omnitrope and the original lyophilized formulations for reconstitution of Omnitrope and Genotropin. Eur. J. Endocrinol. 162 (6), 1051–1058. 10.1530/eje-09-1101 20332125

[B48] GBD 2017 Inflammatory Bowel Disease Collaborators (2020). The global, regional, and national burden of inflammatory bowel disease in 195 countries and territories, 1990-2017: a systematic analysis for the Global Burden of Disease Study 2017. Lancet Gastroenterol. Hepatol. 5 (1), 17–30. 10.1016/s2468-1253(19)30333-4 31648971 PMC7026709

[B49] Generics and Biosimilars Initiative (2023a). Biosimilars approved in Europe. Available at: https://www.gabionline.net/biosimilars/general/biosimilars-approved-in-europe (Accessed March 30, 2024).

[B50] Generics and Biosimilars Initiative (2023b). Biosimilars approved in the US. Available at: https://www.gabionline.net/biosimilars/general/biosimilars-approved-in-the-us (Accessed March 30, 2024).

[B51] Genovese M. C. Sanchez-Burson J. Oh M. Balazs E. Neal J. Everding A. (2020). Comparative clinical efficacy and safety of the proposed biosimilar ABP 710 with infliximab reference product in patients with rheumatoid arthritis. Arthritis Res. Ther. 22 (1), 60. 10.1186/s13075-020-2142-1 32216829 PMC7098142

[B52] Goll G. L. Jørgensen K. K. Sexton J. Olsen I. C. Bolstad N. Haavardsholm E. A. (2019). Long-term efficacy and safety of biosimilar infliximab (CT-P13) after switching from originator infliximab: open-label extension of the NOR-SWITCH trial. J. Intern Med. 285 (6), 653–669. 10.1111/joim.12880 30762274 PMC6850326

[B53] Haifer C. Srinivasan A. An Y. K. Picardo S. van Langenberg D. Menon S. (2021). Switching Australian patients with moderate to severe inflammatory bowel disease from originator to biosimilar infliximab: a multicentre, parallel cohort study. Med. J. Aust. 214 (3), 128–133. 10.5694/mja2.50824 33070332

[B54] Hanauer S. Liedert B. Balser S. Brockstedt E. Moschetti V. Schreiber S. (2021). Safety and efficacy of BI 695501 versus adalimumab reference product in patients with advanced Crohn's disease (VOLTAIRE-CD): a multicentre, randomised, double-blind, phase 3 trial. Lancet Gastroenterol. Hepatol. 6 (10), 816–825. 10.1016/s2468-1253(21)00252-1 34388360

[B55] Hanauer S. B. Feagan B. G. Lichtenstein G. R. Mayer L. F. Schreiber S. Colombel J. F. (2002). Maintenance infliximab for Crohn's disease: the ACCENT I randomised trial. Lancet 359 (9317), 1541–1549. 10.1016/s0140-6736(02)08512-4 12047962

[B56] Hanauer S. B. Sandborn W. J. Rutgeerts P. Fedorak R. N. Lukas M. MacIntosh D. (2006). Human anti-tumor necrosis factor monoclonal antibody (adalimumab) in Crohn's disease: the CLASSIC-I trial. Gastroenterology 130 (2), 323–333. quiz 591. 10.1053/j.gastro.2005.11.030 16472588

[B57] Hanzel J. Jansen J. M. Ter Steege R. W. F. Gecse K. B. D'Haens G. R. (2022). Multiple switches from the originator infliximab to biosimilars is effective and safe in inflammatory bowel disease: a prospective multicenter cohort study. Inflamm. Bowel Dis. 28 (4), 495–501. 10.1093/ibd/izab099 34013959 PMC8972297

[B58] Hu S. Liang S. Guo H. Zhang D. Li H. Wang X. (2013). Comparison of the inhibition mechanisms of adalimumab and infliximab in treating tumor necrosis factor α-associated diseases from a molecular view. J. Biol. Chem. 288 (38), 27059–27067. 10.1074/jbc.M113.491530 23943614 PMC3779706

[B59] IQVIA Institute (2023a). Biosimilars in the United States 2023-2027: competition, savings, and sustainability. Available at: https://www.iqvia.com/insights/the-iqvia-institute/reports-and-publications/reports/biosimilars-in-the-united-states-2023-2027 (Accessed March 30, 2024).

[B60] IQVIA Institute (2023b). The impact of biosimilar competition in Europe. Available at: https://www.iqvia.com/-/media/iqvia/pdfs/library/white-papers/the-impact-of-biosimilar-competition-in-europe-2023.pdf (Accessed March 30, 2023).

[B61] Jang D. I. Lee A. H. Shin H. Y. Song H. R. Park J. H. Kang T. B. (2021). The role of tumor necrosis factor alpha (TNF-α) in autoimmune disease and current TNF-α inhibitors in therapeutics. Int. J. Mol. Sci. 22 (5), 2719. 10.3390/ijms22052719 33800290 PMC7962638

[B62] Jha A. Upton A. Dunlop W. C. Akehurst R. (2015). The budget impact of biosimilar infliximab (Remsima®) for the treatment of autoimmune diseases in five European countries. Adv. Ther. 32 (8), 742–756. 10.1007/s12325-015-0233-1 26343027 PMC4569679

[B63] Jiang F. Wu M. Li R. (2023). The significance of long non-coding RNAs in the pathogenesis, diagnosis and treatment of inflammatory bowel disease. Precis. Clin. Med. 6 (4), pbad031. 10.1093/pcmedi/pbad031 38163004 PMC10757071

[B64] Jiang M. Zeng Z. Chen K. Dang Y. Li L. Ma C. (2022). Enterogenous microbiotic markers in the differential diagnosis of crohn's disease and intestinal tuberculosis. Front. Immunol. 13, 820891. 10.3389/fimmu.2022.820891 35371004 PMC8966387

[B65] Jin R. Nduka C. Courmier D. Knight H. Meadows R. Piercy J. (2024). Real-world experience of adalimumab biosimilar (ABP 501) use in patients with inflammatory bowel disease in Europe. Adv. Ther. 41 (1), 331–348. 10.1007/s12325-023-02712-w 37957522 PMC10796661

[B66] Jørgensen K. K. Olsen I. C. Goll G. L. Lorentzen M. Bolstad N. Haavardsholm E. A. (2017). Switching from originator infliximab to biosimilar CT-P13 compared with maintained treatment with originator infliximab (NOR-SWITCH): a 52-week, randomised, double-blind, non-inferiority trial. Lancet 389 (10086), 2304–2316. 10.1016/s0140-6736(17)30068-5 28502609

[B67] Kang H. N. Wadhwa M. Knezevic I. Ondari C. Simao M. (2023). WHO guidelines on biosimilars: toward improved access to safe and effective products. Ann. N. Y. Acad. Sci. 1521 (1), 96–103. 10.1111/nyas.14965 36694455

[B68] Kaplan G. G. Windsor J. W. (2021). The four epidemiological stages in the global evolution of inflammatory bowel disease. Nat. Rev. Gastroenterol. Hepatol. 18 (1), 56–66. 10.1038/s41575-020-00360-x 33033392 PMC7542092

[B69] Kappelman M. D. Rifas-Shiman S. L. Porter C. Q. Ollendorf D. A. Sandler R. S. Galanko J. A. (2008). Direct health care costs of Crohn's disease and ulcerative colitis in US children and adults. Gastroenterology 135 (6), 1907–1913. 10.1053/j.gastro.2008.09.012 18854185 PMC2613430

[B70] Kaur P. Chow V. Zhang N. Moxness M. Kaliyaperumal A. Markus R. (2017). A randomised, single-blind, single-dose, three-arm, parallel-group study in healthy subjects to demonstrate pharmacokinetic equivalence of ABP 501 and adalimumab. Ann. Rheum. Dis. 76 (3), 526–533. 10.1136/annrheumdis-2015-208914 27466231 PMC5445997

[B71] Kay J. Cross R. K. Feldman S. R. Park Y. Hanauer S. B. (2024). Review of adalimumab biosimilar SB5 in immune-mediated inflammatory diseases. Adv. Ther. 41 (2), 509–533. 10.1007/s12325-023-02737-1 38110655 PMC10838831

[B72] Kennedy N. A. Heap G. A. Green H. D. Hamilton B. Bewshea C. Walker G. J. (2019). Predictors of anti-TNF treatment failure in anti-TNF-naive patients with active luminal Crohn's disease: a prospective, multicentre, cohort study. Lancet Gastroenterol. Hepatol. 4 (5), 341–353. 10.1016/s2468-1253(19)30012-3 30824404

[B73] Knight D. M. Trinh H. Le J. Siegel S. Shealy D. McDonough M. (1993). Construction and initial characterization of a mouse-human chimeric anti-TNF antibody. Mol. Immunol. 30 (16), 1443–1453. 10.1016/0161-5890(93)90106-l 8232330

[B74] Kvien T. K. Patel K. Strand V. (2022). The cost savings of biosimilars can help increase patient access and lift the financial burden of health care systems. Semin. Arthritis Rheum. 52, 151939. 10.1016/j.semarthrit.2021.11.009 35027243

[B75] Laharie D. Bourreille A. Branche J. Allez M. Bouhnik Y. Filippi J. (2021). Evolution of endoscopic lesions in steroid-refractory acute severe ulcerative colitis responding to infliximab or cyclosporine. Clin. Gastroenterol. Hepatol. 19 (6), 1180–1188.e4. 10.1016/j.cgh.2020.08.001 32777552

[B76] Lambert J. Wyand M. Lassen C. Shneyer L. Thomson E. Knight A. (2016). Bioavailability, safety and immunogenicity of biosimilar infliximab (BOW015) compared to reference infliximab. Int. J. Clin. Pharmacol. Ther. 54 (4), 315–322. 10.5414/cp202530 26952037

[B77] Lee Y. H. Song G. G. (2023). Comparative efficacy and safety of infliximab and its biosimilars in patients with rheumatoid arthritis presenting an insufficient response to methotrexate: a network meta-analysis. Z Rheumatol. 82 (2), 114–122. 10.1007/s00393-021-01040-0 34228181

[B78] Leone G. M. Mangano K. Petralia M. C. Nicoletti F. Fagone P. (2023). Past, present and (foreseeable) future of biological anti-TNF alpha therapy. J. Clin. Med. 12 (4), 1630. 10.3390/jcm12041630 36836166 PMC9963154

[B79] Levin A. D. Wildenberg M. E. van den Brink G. R. (2016). Mechanism of action of anti-TNF therapy in inflammatory bowel disease. J. Crohns Colitis 10 (8), 989–997. 10.1093/ecco-jcc/jjw053 26896086

[B80] Liu Y. Liu S. Liu L. Gong X. Liu J. Sun L. (2022). Fine comparison of the efficacy and safety between GB242 and infliximab in patients with rheumatoid arthritis: a phase III study. Rheumatol. Ther. 9 (1), 175–189. 10.1007/s40744-021-00396-8 34806155 PMC8814292

[B81] Luber R. P. O'Neill R. Singh S. Sharma E. Cunningham G. Honap S. (2021). An observational study of switching infliximab biosimilar: no adverse impact on inflammatory bowel disease control or drug levels with first or second switch. Aliment. Pharmacol. Ther. 54 (5), 678–688. 10.1111/apt.16497 34223654

[B82] Lukas M. Malickova K. Kolar M. Bortlik M. Vasatko M. Machkova N. (2020). Switching from originator adalimumab to the biosimilar SB5 in patients with inflammatory bowel disease: short-term experience from a single tertiary clinical centre. J. Crohns Colitis 14 (7), 915–919. 10.1093/ecco-jcc/jjaa001 31905382

[B83] Lyman G. H. Zon R. Harvey R. D. Schilsky R. L. (2018). Rationale, opportunities, and reality of biosimilar medications. N. Engl. J. Med. 378 (21), 2036–2044. 10.1056/NEJMhle1800125 29791832

[B84] Macaluso F. S. Cappello M. Busacca A. Fries W. Viola A. Costantino G. (2021a). SPOSAB ABP 501: a Sicilian prospective observational study of patients with inflammatory bowel disease treated with adalimumab biosimilar ABP 501. J. Gastroenterol. Hepatol. 36 (11), 3041–3049. 10.1111/jgh.15590 34152636

[B85] Macaluso F. S. Casà A. Renna S. Grova M. Mannino M. Orlando A. (2023). Switching from SB2 to PF-06438179/GP1111 and back in inflammatory bowel disease: "The Superswitchers. Dig. Liver Dis. 55 (3), 424–425. 10.1016/j.dld.2022.12.008 36609013

[B86] Macaluso F. S. Fries W. Viola A. Centritto A. Cappello M. Giuffrida E. (2021b). The SPOSIB SB2 Sicilian cohort: safety and effectiveness of infliximab biosimilar SB2 in inflammatory bowel diseases, including multiple switches. Inflamm. Bowel Dis. 27 (2), 182–189. 10.1093/ibd/izaa036 32083291

[B87] Massimi D. Barberio B. Bertani L. Costa F. Ferronato A. Facchin S. (2021). Switching from infliximab originator to SB2 biosimilar in inflammatory bowel diseases: a multicentric prospective real-life study. Ther. Adv. Gastroenterol. 14, 17562848211023384. 10.1177/17562848211023384 PMC8239954 34249147

[B88] Menter A. Cohen S. Kay J. Strand V. Gottlieb A. Hanauer S. (2022). Switching between adalimumab reference product and BI 695501 in patients with chronic plaque psoriasis (VOLTAIRE-X): a randomized controlled trial. Am. J. Clin. Dermatol 23 (5), 719–728. 10.1007/s40257-022-00708-w 35934770 PMC9464749

[B89] Meyer A. Rudant J. Drouin J. Weill A. Carbonnel F. Coste J. (2019). Effectiveness and safety of reference infliximab and biosimilar in crohn disease: a French equivalence study. Ann. Intern Med. 170 (2), 99–107. 10.7326/m18-1512 30534946

[B90] Mocci G. Bodini G. Allegretta L. Cazzato A. I. Chiri S. Aragona G. (2022). Adalimumab biosimilar GP2017 versus adalimumab originator in treating patients with inflammatory bowel diseases: a real-life, multicenter, observational study. Biomedicines 10 (8), 1799. 10.3390/biomedicines10081799 35892698 PMC9331541

[B91] Müller-Ladner U. Dignass A. Gaffney K. Jadon D. Matucci-Cerinic M. Lobaton T. (2023). The proper study: a 48-week, pan-European, real-world study of biosimilar SB5 following transition from reference adalimumab in patients with immune-mediated inflammatory disease. BioDrugs 37 (6), 873–889. 10.1007/s40259-023-00616-3 37632666 PMC10581927

[B92] Odes S. Vardi H. Friger M. Wolters F. Russel M. G. Riis L. (2006). Cost analysis and cost determinants in a European inflammatory bowel disease inception cohort with 10 years of follow-up evaluation. Gastroenterology 131 (3), 719–728. 10.1053/j.gastro.2006.05.052 16952541

[B93] Palaparthy R. Udata C. Hua S. Y. Yin D. Cai C. H. Salts S. (2018). A randomized study comparing the pharmacokinetics of the potential biosimilar PF-06438179/GP1111 with Remicade® (infliximab) in healthy subjects (REFLECTIONS B537-01). Expert Rev. Clin. Immunol. 14 (4), 329–336. 10.1080/1744666x.2018.1446829 29504427

[B94] Papp K. Bachelez H. Costanzo A. Foley P. Gooderham M. Kaur P. (2017). Clinical similarity of biosimilar ABP 501 to adalimumab in the treatment of patients with moderate to severe plaque psoriasis: a randomized, double-blind, multicenter, phase III study. J. Am. Acad. Dermatol 76 (6), 1093–1102. 10.1016/j.jaad.2016.12.014 28291552

[B95] Parigi T. L. D'Amico F. Peyrin-Biroulet L. Danese S. (2021). Evolution of infliximab biosimilar in inflammatory bowel disease: from intravenous to subcutaneous CT-P13. Expert Opin. Biol. Ther. 21 (1), 37–46. 10.1080/14712598.2020.1811849 32799561

[B96] Park K. T. Ehrlich O. G. Allen J. I. Meadows P. Szigethy E. M. Henrichsen K. (2020). The cost of inflammatory bowel disease: an initiative from the crohn's and colitis foundation. Inflamm. Bowel Dis. 26 (1), 1–10. 10.1093/ibd/izz104 31112238 PMC7534391

[B97] Park W. Hrycaj P. Jeka S. Kovalenko V. Lysenko G. Miranda P. (2013). A randomised, double-blind, multicentre, parallel-group, prospective study comparing the pharmacokinetics, safety, and efficacy of CT-P13 and innovator infliximab in patients with ankylosing spondylitis: the PLANETAS study. Ann. Rheum. Dis. 72 (10), 1605–1612. 10.1136/annrheumdis-2012-203091 23687259 PMC3786643

[B98] Reinisch W. Jang B. I. Borzan V. Lahat A. Pukitis A. Osipenko M. (2019). DOP62 A novel formulation of CT-P13 (infliximab biosimilar) for subcutaneous administration: 1-year result from a Phase I open-label randomised controlled trial in patients with active Crohn’s disease. J. Crohn's Colitis 13 (Suppl. ment_1), S066–S067. 10.1093/ecco-jcc/jjy222.096

[B99] Reinisch W. Sandborn W. J. Hommes D. W. D'Haens G. Hanauer S. Schreiber S. (2011). Adalimumab for induction of clinical remission in moderately to severely active ulcerative colitis: results of a randomised controlled trial. Gut 60 (6), 780–787. 10.1136/gut.2010.221127 21209123

[B100] Ribaldone D. G. Caviglia G. P. Pellicano R. Vernero M. Saracco G. M. Morino M. (2020). Effectiveness and safety of adalimumab biosimilar ABP 501 in Crohn's disease: an observational study. Rev. Esp. Enferm. Dig. 112 (3), 195–200. 10.17235/reed.2020.6693/2019 32054272

[B101] Ribaldone D. G. Tribocco E. Rosso C. Armandi A. Vernero M. Bugianesi E. (2021). Switching from biosimilar to biosimilar adalimumab, including multiple switching, in crohn's disease: a prospective study. J. Clin. Med. 10 (15), 3387. 10.3390/jcm10153387 34362184 PMC8348781

[B102] Rutgeerts P. Sandborn W. J. Feagan B. G. Reinisch W. Olson A. Johanns J. (2005). Infliximab for induction and maintenance therapy for ulcerative colitis. N. Engl. J. Med. 353 (23), 2462–2476. 10.1056/NEJMoa050516 16339095

[B103] Saleem R. Cantin G. Wikström M. Bolton G. Kuhns S. McBride H. J. (2020). Analytical and functional similarity assessment of ABP 710, a biosimilar to infliximab reference product. Pharm. Res. 37 (6), 114. 10.1007/s11095-020-02816-w 32476063 PMC7261735

[B104] Sandborn W. J. Hanauer S. B. Rutgeerts P. Fedorak R. N. Lukas M. MacIntosh D. G. (2007). Adalimumab for maintenance treatment of Crohn's disease: results of the CLASSIC II trial. Gut 56 (9), 1232–1239. 10.1136/gut.2006.106781 17299059 PMC2701613

[B105] Sandborn W. J. van Assche G. Reinisch W. Colombel J. F. D'Haens G. Wolf D. C. (2012). Adalimumab induces and maintains clinical remission in patients with moderate-to-severe ulcerative colitis. Gastroenterology 142 (2), 257–265. 10.1053/j.gastro.2011.10.032 22062358

[B106] Sands B. E. Anderson F. H. Bernstein C. N. Chey W. Y. Feagan B. G. Fedorak R. N. (2004). Infliximab maintenance therapy for fistulizing Crohn's disease. N. Engl. J. Med. 350 (9), 876–885. 10.1056/NEJMoa030815 14985485

[B107] San-Juan-Rodriguez A. Gellad W. F. Good C. B. Hernandez I. (2019). Trends in list prices, net prices, and discounts for originator biologics facing biosimilar competition. JAMA Netw. Open 2 (12), e1917379. 10.1001/jamanetworkopen.2019.17379 31834391 PMC6938676

[B108] Sarlós P. Bikar A. Farkas N. Resál T. Szepes Z. Farkas K. (2023). Self-reported efficacy and safety of infliximab and adalimumab biosimilars after non-medical switch in patients with inflammatory bowel disease: results of a multicenter survey. Expert Opin. Biol. Ther. 23 (8), 827–832. 10.1080/14712598.2023.2211204 37161387

[B109] Schmitz E. M. H. Boekema P. J. Straathof J. W. A. van Renswouw D. C. Brunsveld L. Scharnhorst V. (2018). Switching from infliximab innovator to biosimilar in patients with inflammatory bowel disease: a 12-month multicentre observational prospective cohort study. Aliment. Pharmacol. Ther. 47 (3), 356–363. 10.1111/apt.14453 29205444

[B110] Schreiber S. Ben-Horin S. Leszczyszyn J. Dudkowiak R. Lahat A. Gawdis-Wojnarska B. (2021). Randomized controlled trial: subcutaneous vs intravenous infliximab CT-P13 maintenance in inflammatory bowel disease. Gastroenterology 160 (7), 2340–2353. 10.1053/j.gastro.2021.02.068 33676969

[B111] Shin D. Kim Y. Kim Y. S. Körnicke T. Fuhr R. (2015). A randomized, phase I pharmacokinetic study comparing SB2 and infliximab reference product (Remicade(®)) in healthy subjects. BioDrugs 29 (6), 381–388. 10.1007/s40259-015-0150-5 26577771 PMC4684585

[B112] Singh S. Murad M. H. Fumery M. Sedano R. Jairath V. Panaccione R. (2021). Comparative efficacy and safety of biologic therapies for moderate-to-severe Crohn's disease: a systematic review and network meta-analysis. Lancet Gastroenterol. Hepatol. 6 (12), 1002–1014. 10.1016/s2468-1253(21)00312-5 34688373 PMC8933137

[B113] Smith P. J. Critchley L. Storey D. Gregg B. Stenson J. Kneebone A. (2022). Efficacy and safety of elective switching from intravenous to subcutaneous infliximab [ct-P13]: a multicentre cohort study. J. Crohns Colitis 16 (9), 1436–1446. 10.1093/ecco-jcc/jjac053 35390141 PMC9455786

[B114] Soubières A. A. Poullis A. (2016). Emerging biomarkers for the diagnosis and monitoring of inflammatory bowel diseases. Inflamm. Bowel Dis. 22 (8), 2016–2022. 10.1097/mib.0000000000000836 27416044

[B115] Strand V. Gonçalves J. Hickling T. P. Jones H. E. Marshall L. Isaacs J. D. (2020). Immunogenicity of biosimilars for rheumatic diseases, plaque psoriasis, and inflammatory bowel disease: a review from clinical trials and regulatory documents. BioDrugs 34 (1), 27–37. 10.1007/s40259-019-00394-x 31721107 PMC7042210

[B116] Strik A. S. van de Vrie W. Bloemsaat-Minekus J. P. J. Nurmohamed M. Bossuyt P. J. J. Bodelier A. (2018). Serum concentrations after switching from originator infliximab to the biosimilar CT-P13 in patients with quiescent inflammatory bowel disease (SECURE): an open-label, multicentre, phase 4 non-inferiority trial. Lancet Gastroenterol. Hepatol. 3 (6), 404–412. 10.1016/s2468-1253(18)30082-7 29606564

[B117] Su C. G. Lichtenstein G. R. (2003). Influence of immunogenicity on the long-term efficacy of infliximab in Crohn's disease. Gastroenterology 125 (5), 1544–1546. 10.1016/j.gastro.2003.05.009 14598275

[B118] Tapete G. Bertani L. Pieraccini A. Lynch E. N. Giannotta M. Morganti R. (2022). Effectiveness and safety of nonmedical switch from adalimumab originator to SB5 biosimilar in patients with inflammatory bowel diseases: twelve-month follow-up from the TABLET registry. Inflamm. Bowel Dis. 28 (1), 62–69. 10.1093/ibd/izab027 33570142

[B119] Tracey D. Klareskog L. Sasso E. H. Salfeld J. G. Tak P. P. (2008). Tumor necrosis factor antagonist mechanisms of action: a comprehensive review. Pharmacol. Ther. 117 (2), 244–279. 10.1016/j.pharmthera.2007.10.001 18155297

[B120] Tursi A. Mocci G. Allegretta L. Aragona G. Bianco M. A. Colucci R. (2023). Comparison of performances of adalimumab biosimilars SB5, ABP501, GP2017, and MSB11022 in treating patients with inflammatory bowel diseases: a real-life, multicenter, observational study. Inflamm. Bowel Dis. 29 (3), 376–383. 10.1093/ibd/izac092 35579320

[B121] Tursi A. Mocci G. Cuomo A. Ferronato A. Elisei W. Picchio M. (2022). Replacement of adalimumab originator to adalimumab biosimilar for a non-medical reason in patients with inflammatory bowel disease: a real-life comparison of adalimumab biosimilars currently available in Italy. J. Gastrointestin Liver Dis. 31 (4), 411–416. 10.15403/jgld-4608 36535057

[B122] van der Valk M. E. Mangen M. J. Leenders M. Dijkstra G. van Bodegraven A. A. Fidder H. H. (2014). Healthcare costs of inflammatory bowel disease have shifted from hospitalisation and surgery towards anti-TNFα therapy: results from the COIN study. Gut 63 (1), 72–79. 10.1136/gutjnl-2012-303376 23135759

[B123] Vernero M. Bezzio C. Ribaldone D. G. Costa S. Scalvini D. Tribocco E. (2023). Efficacy and safety of adalimumab biosimilar GP2017 in patients with inflammatory bowel disease. J. Clin. Med. 12 (21), 6839. 10.3390/jcm12216839 37959304 PMC10647534

[B124] Wang B. Shen J. (2022). NF-κB inducing kinase regulates intestinal immunity and homeostasis. Front. Immunol. 13, 895636. 10.3389/fimmu.2022.895636 35833111 PMC9271571

[B125] Wasserbauer M. Hlava S. Drabek J. Stovicek J. Minarikova P. Nedbalova L. (2022). Adalimumab biosimilars in the therapy of Crohn´s disease and ulcerative colitis: prospective multicentric clinical monitoring. PLoS One 17 (8), e0271299. 10.1371/journal.pone.0271299 35939424 PMC9359532

[B126] Weinblatt M. E. Baranauskaite A. Niebrzydowski J. Dokoupilova E. Zielinska A. Jaworski J. (2018). Phase III randomized study of SB5, an adalimumab biosimilar, versus reference adalimumab in patients with moderate-to-severe rheumatoid arthritis. Arthritis Rheumatol. 70 (1), 40–48. 10.1002/art.40336 28950421 PMC5765475

[B127] Wiland P. Jeka S. Dokoupilová E. Brandt-Jürgens J. Miranda Limón J. M. Cantalejo Moreira M. (2020). Switching to biosimilar SDZ-ADL in patients with moderate-to-severe active rheumatoid arthritis: 48-week efficacy, safety and immunogenicity results from the phase III, randomized, double-blind ADMYRA study. BioDrugs 34 (6), 809–823. 10.1007/s40259-020-00447-6 33119861 PMC7669771

[B128] Wilson M. R. Bergman A. Chevrou-Severac H. Selby R. Smyth M. Kerrigan M. C. (2018). Cost-effectiveness of vedolizumab compared with infliximab, adalimumab, and golimumab in patients with ulcerative colitis in the United Kingdom. Eur. J. Health Econ. 19 (2), 229–240. 10.1007/s10198-017-0879-5 28271250

[B129] World Health Organization (2022). Guidelines on evaluation of biosimilars. Replacement of annex 2 of WHO technical report series, No. 977. Geneva: World Health Organization. Available at: https://www.who.int/publications/m/item/guidelines-on-evaluation-of-biosimilars (Accessed March 30, 2024).

[B130] Wynne C. Altendorfer M. Sonderegger I. Gheyle L. Ellis-Pegler R. Buschke S. (2016). Bioequivalence, safety and immunogenicity of BI 695501, an adalimumab biosimilar candidate, compared with the reference biologic in a randomized, double-blind, active comparator phase I clinical study (VOLTAIRE®-PK) in healthy subjects. Expert Opin. Investig. Drugs 25 (12), 1361–1370. 10.1080/13543784.2016.1255724 27813422

[B131] Xu B. C. Li X. J. Gao M. Y. (2021). Influence of commercial insurance purchase on the health status of Chinese residents. Front. Public Health 9, 752530. 10.3389/fpubh.2021.752530 34604168 PMC8481586

[B132] Yamamoto S. Nakase H. Matsuura M. Honzawa Y. Masuda S. Inui K. (2010). Efficacy and safety of infliximab as rescue therapy for ulcerative colitis refractory to tacrolimus. J. Gastroenterol. Hepatol. 25 (5), 886–891. 10.1111/j.1440-1746.2009.06206.x 20546441

[B133] Ye B. D. Pesegova M. Alexeeva O. Osipenko M. Lahat A. Dorofeyev A. (2019). Efficacy and safety of biosimilar CT-P13 compared with originator infliximab in patients with active Crohn's disease: an international, randomised, double-blind, phase 3 non-inferiority study. Lancet 393 (10182), 1699–1707. 10.1016/s0140-6736(18)32196-2 30929895

[B134] Ye H. Liu S. Xu J. Chai K. He D. Fang Y. (2023). Efficacy and safety of CMAB008 compared with innovator infliximab in patients with moderate-to-severe rheumatoid arthritis receiving concomitant methotrexate: a randomized, double-blind, multi-center, phase III non-inferiority study. Rheumatol. Ther. 10 (3), 757–773. 10.1007/s40744-023-00544-2 36964872 PMC10140208

[B135] Yoo D. H. Hrycaj P. Miranda P. Ramiterre E. Piotrowski M. Shevchuk S. (2013). A randomised, double-blind, parallel-group study to demonstrate equivalence in efficacy and safety of CT-P13 compared with innovator infliximab when coadministered with methotrexate in patients with active rheumatoid arthritis: the PLANETRA study. Ann. Rheum. Dis. 72 (10), 1613–1620. 10.1136/annrheumdis-2012-203090 23687260 PMC3786641

[B136] Yu Q. Zhu C. Feng S. Xu L. Hu S. Chen H. (2021). Economic burden and health care access for patients with inflammatory bowel diseases in China: web-based survey study. J. Med. Internet Res. 23 (1), e20629. 10.2196/20629 33399540 PMC7815453

[B137] Yueying C. Jing F. Qi F. Jun S. (2023). Infliximab response associates with radiologic findings in bio-naïve Crohn's disease. Eur. Radiol. 33 (8), 5247–5257. 10.1007/s00330-023-09542-y 36928565 PMC10326128

[B138] Zeng Z. Jiang M. Li X. Yuan J. Zhang H. (2023). Precision medicine in inflammatory bowel disease. Precis. Clin. Med. 6 (4), pbad033. 10.1093/pcmedi/pbad033 38638127 PMC11025389

[B139] Zhang T. Chen G. Liu C. Zu L. Wang Q. Wang Y. (2019). A phase I study comparing the pharmacokinetics, safety, and immunogenicity of proposed biosimilar GB242 and reference infliximab in healthy subjects. BioDrugs 33 (1), 93–100. 10.1007/s40259-018-0326-x 30511316

[B140] Zhao M. Gönczi L. Lakatos P. L. Burisch J. (2021). The burden of inflammatory bowel disease in Europe in 2020. J. Crohns Colitis 15 (9), 1573–1587. 10.1093/ecco-jcc/jjab029 33582812

[B141] Zheng M. K. Shih D. Q. Chen G. C. (2017). Insights on the use of biosimilars in the treatment of inflammatory bowel disease. World J. Gastroenterol. 23 (11), 1932–1943. 10.3748/wjg.v23.i11.1932 28373759 PMC5360634

