# Supplementary material for: Anti-TNFα in inflammatory bowel disease: from originators to biosimilars
Source: Front Pharmacol. 2024 Jul 24;15:1424606. doi: 10.3389/fphar.2024.1424606 (PMC11303209; doi:10.3389/fphar.2024.1424606)
Supplement: Supplementary file 1 [file Table1.DOCX]

Supplementary Material

**
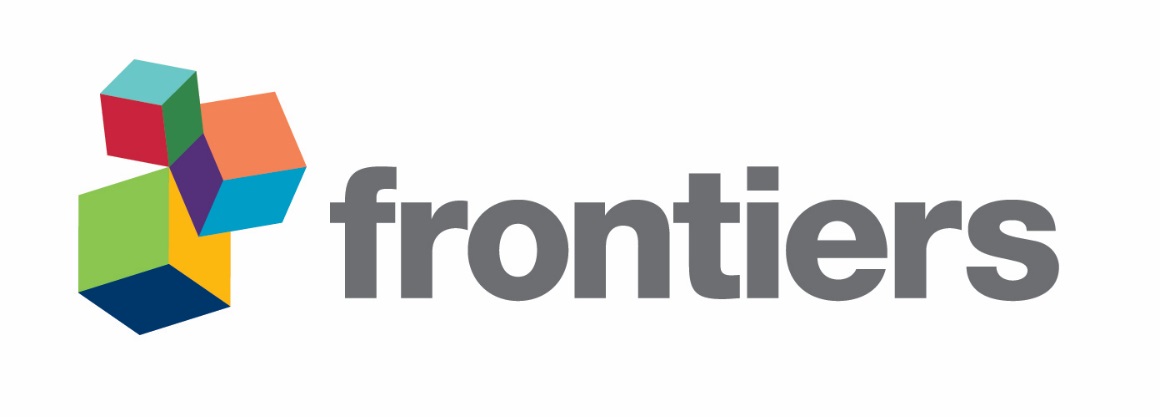
**

**Table S1.** **Clinical trials of infliximab biosimilars in inflammatory bowel disease**

| **Originator** | **Biosimilar** | **Study design** | **Population** | **Subgroup** | **Efficacy** | **Safety** | **Pharmacokinetics** | **Immunogenicity** | **Reference** |
| --- | --- | --- | --- | --- | --- | --- | --- | --- | --- |
| IFX | CT-P13 (IV formulation) | Prospective, multicenter, cohort study | CD=313  UC=234 | 1. Naive to anti-TNFα  2.Previous exposed to anti-TNFα  3.Switch from IFX to CT-P13 | The clinical response rates at weeks 8,16, and 24 were 95.7%, 86.4%, and 73.7% for naive, 97.2%, 85.2%, and 62.2% for pre-exposed, and 94.5%, 90.8%, and 78.9% for switch, respectively. | SAEs: 66 (12.1%)  Infusion-related reaction: 38 (6.9%) | N/A | N/A | (Fiorino et al., 2017) |
| IFX | CT-P13 (IV formulation) | Comparative, equivalence, cohort study | CD=5050 | 1.IFX treatment group  2. CT-P13 treatment group | CT-P13 was equivalent to IFX in terms of death, CD-related surgery, all-cause hospitalization, and reimbursement of another biologic therapy (HR, 0.92 [95% CI, 0.85 to 0.99]) | No differences in safety outcomes: serious infections, tuberculosis, and solid or hematologic cancer. | N/A | N/A | (Meyer et al., 2019) |
| IFX | CT-P13 (IV formulation) | Prospective, open label, multicenter, parallel cohort, non-inferiority study | CD=232  UC=113 | 1. IFX maintenance treatment group (control)  2. CT-P13 switching treatment group (switch) | The clinical deterioration rates (7% vs. 8%) and therapeutic discontinuation rates (14% vs. 15%) were similar between the control and the switch group. | SAEs leading to discontinuation in the control group (6/141, 4%) and the switch group (6/204, 3%) were similar. | Similar C_trough_ in the two groups at all time points. | N/A | (Haifer et al., 2021) |
| IFX | CT-P13 (IV formulation) | Multicenter, observational, prospective, cohort study | CD=86  UC=47 | 1. Before CT-P13 switching treatment  2. After CT-P13 switching treatment | CRP levels and disease activity scores were not significantly different before and after switching. | AEs: 9.8% | C_trough_ before switching: 3.5 μg/mL.  C_trough_ after switching: 3.5 to 4.2 μg/mL | Three patients developed ADAbs after switching. | (Schmitz et al., 2018) |
| IFX | CT-P13 (IV formulation) | Randomized, multicenter, double-blind, phase III non-inferiority study | CD=220 | 1. CT-P13-CT-P13 group  2. CT-P13-IFX group  3. IFX-IFX group  4. IFX-CT-P13 group | CDAI-70 response rates at weeks 6, 14, and 30 were similar for CT-P13 (69·4%, 86.5%, and 76.6%, respectively) and IFX (74·3%, 88.1%, and 75.2%, respectively). | All TEAEs: 147 (67%);  The CT-P13-CT-P13 group: 36 (64%); The CT-P13-IFX group: 34 (62%);  The IFX-IFX group: 37 (69%);  The IFX-CT-P13 group: 40 (73%). | C_max_ and C_trough_ values in CT-P13 and IFX groups were similar at weeks 0, 2, 6, and 14. | ADAbs was similar between the CT-P13 and IFX treatment groups at week 14 (14% vs. 17%) and  week 54 (39% vs. 33%). | (Ye et al., 2019) |
| IFX | CT-P13 (IV formulation) | Randomized, non-inferiority, double-blind, phase IV trial | CD=155  UC=93  Spondylarthritis =91  RA=77  Psoriatic arthritis=30  Psoriasis=35 | 1. IFX maintenance treatment group  2. CT-P13 switching treatment group | Disease worsening occurred in 53 (26%) patients in the IFX group and 61 (30%) patients in the CT-P13 switching treatment group | The overall AEs (70% vs. 68%) and SAEs (10% vs. 9%) were similar between the IFX group and the CT-P13 switching group. | Similar C_trough_ in the two groups during follow-up. | The incidence of ADAbs was 7% for IFX and 8% for CT-P13. | (Jørgensen et al., 2017) |
| IFX | CT-P13 (IV formulation) | Prospective, open-label, interventional, non-inferiority, multicenter, phase IV trial | CD=61  UC=59 | 1. Before CT-P13 switching treatment  2. After CT-P13 switching treatment | Median CRP levels (at weeks 8 and 16) and FC concentrations (at week 16) were not significantly different before and after switching. | TEAEs were 64 (CD 34, UC 30); SAEs were 8 (CD 6, UC 8). | The geometric mean ratio of Ctrough at week 16 was 110.1% (CT-P13 vs. IFX, 90% CI 96.0-126.3) in UC and 107·6% (97.4-118.8) in CD. | Three patients developed new ADAbs after switching (at 16 weeks) | (Strik et al., 2018) |
| IFX | CT-P13 (IV formulation) | Retrospective, cohort, multicenter study | CD=353  UC=123 | 1. Non-switch cohort (NC)  2. Switch cohort (SC) | The incidence of relapse in the NC and the SC was 5% and 14% per patient-year, respectively. The switch to CT-P13 was associated with a higher risk of relapse (HR = 3.5, 95% CI = 2-6). | AEs were higher in the NC (30%), compared with 6% in the SC. | N/A | N/A | (Chaparro et al., 2019) |
| IFX | CT-P13 (IV and SC formulation) | Open-label, randomized, multicenter, parallel group phase I study | CD=53  UC=78 | 1. CT-P13 IV treatment group  2. CT-P13 SC treatment group | The clinical response rates (86.8% vs. 74.4%, at week 30), clinical remission rates (60.5% vs. 38.5%, at week 30), and mucosal healing rates (47.7% vs. 30.8%, at week 22) were comparable between the CT-P13 SC group and the CT-P13 IV group. | TEAEs in the CT-P13 SC: 38 (57.6%); TEAEs in the CT-P13 IV: 32 (49.2%) | Mean Ctrough at week 22 was higher in the CT-P13 SC arm than in the CT-P13 IV arm (21.45 mg/mL vs 2.93 mg/mL). | ADAbs were similar between the groups up to Week30. | (Schreiber et al., 2021) |
| IFX | CT-P13 (IV and SC) | Phase I open-label, randomized, controlled trial | CD=44 | 1. CT-P13 IV treatment group  2. CT-P13 SC treatment group | The clinical remission rates were comparable between the IV (5mg/kg) and the SC cohorts (120mg, 180mg, and 240mg) at weeks 6 (25% vs. 54.5% vs. 16.7% vs. 14.3%), 22 (41.7% vs. 63.6% vs. 33.3% vs. 42.9%), and 30 (58.3% vs. 81.8% vs. 58.3% vs. 71.4%). | Injection site reactions: 11.4% | The mean C_trough_ in the SC cohorts were higher than IV cohort. | N/A | (Reinisch et al., 2019) |
| IFX | CT-P13 (SC formulation) | Retrospective, multicenter, cohort study | CD=115  UC=60  IBD-U=4 | 1. Before CT-P13 switching treatment  2. After CT-P13 switching treatment | The HBI scores (1.0 [2.0] vs. 0.0 [1.0]), SCCAI scores (1.0 [3.0] vs. 0.0 [4.0]), CRP levels (4.0 [2.0] vs. 2.0 [1.0]), and FC concentrations (67.5 [143.5] vs. 79.0 [138.50]) were not significantly different before and at 12 months after switching. | No SAEs were reported. | The mean C_trough_ increased from a baseline of 8.9 µg/dl to 16.0 µg/dl at 3 months. | 14 (7.7%) patients developed new ADAbs after switching. | (Smith et al., 2022) |
| IFX | SB2 | Multicenter, observational, prospective | CD=136  UC=140 | 1. Naïve to IFX and anti-TNFs  2. Naïve to IFX and exposed to anti-TNFs  3. Switch from IFX to SB2  4. Switch from CT-P13 to SB2  5.Switchfrom IFX to CT-P13 to SB2 | 110 patients (110/192, 57.3%) had steroid-free remission, 26 patients (26/192, 13.5%) achieved a partial response, and 56 patients had no response (56/192, 29.2%) after 8 weeks of treatment of SB2 | Total SAEs 20.7%  Infusion reactions: 10.1%  Infections: 3.6% | N/A | N/A | (Macaluso et al., 2021b) |
| IFX | SB2 | Multicentric, prospective, real-life Study | CD=57  UC=28 | 1. Before SB2 switching treatment  2. After SB2 switching treatment | The clinical remission rates (82.3% vs. 69.4%) and FC levels (68.5 µg/g vs. 57.0 µg/g) were not significantly different before and after switching. | No SAEs were recorded. | N/A | No new ADAbs developed. | (Massimi et al., 2021) |
| IFX | SB2 | Prospective, single-center, longitudinal, observational study | CD=94  UC=50 | 1. Before SB2 switching treatment  2. After SB2 switching treatment | The mean changes of disease activity compared with baseline were -0.9 (SD 2.6), -0.4 (2.2), and -0.4 (2.0) in CD and 0.1 (1.1), 0.1 (1.1), and 0.1 (1.3) in UC patients at weeks 24, 48 and 72. | SAE: 7.6%;  AE: 20.1% | The mean C_trough_ were 6.2 µg/ml, 5.0 µg/ml, 6.6 µg/ml, and 5.1 µg/ml at baseline and weeks 24, 48, and 72 | 9.8% of patients developed new ADAbs after switching. | (Fischer et al., 2021) |
| IFX | CT-P13 (IV formulation) and SB2 | Single-center, prospective, observational, cohort study | CD=151  UC=35 | 1. First switch group (from CT-P13 to SB2)  2. Second switch group (from IFX to CT-P13 to SB2) | The clinical remission rates in the first-switch (91% vs. 92%) and the second-switch group (91% vs. 95%) were not significantly different before and after switching. | N/A | The mean C_trough_ were stable in the first-switch (baseline vs early vs. 1year: 5.7 vs. 6.6 vs. 5.7 µg/mL) and the second-switch group (4.3 vs. 4.9 vs. 4.7 µg/mL). | 3.8% of patients developed new ADAbs after the first switching. | (Luber et al., 2021) |
| IFX | CT-P13 (IV formulation) and SB2 | Prospective, multicenter, cohort study | CD=125  UC=49  IBD-U=2 | 1. Switches from IFX to CT-P13 to SB2 (group 1)  2. Switch from CT-P13 to SB2 (group 2)  3. Switch from IFX to CT-P13 (group 3) | The clinical remission rates at 12 months were 76.9% (40/52, group 1), 65.7% (46/70, group 2), and 76.9% (20/26, group 3), respectively. The rates of clinical remission, CRP remission, FC remission, and treatment persistence at 12 months were not significantly different between the three groups. | Infusion reactions: 1.7% | N/A | New ADAbs: 0 in group 1, 3 (3/80, 3.8%) in group 2, 1 (1/27, 3.7%) in group 3. | (Hanzel et al., 2022) |

**Abbreviation:** IFX: infliximab; CD: crohn’s disease; UC: ulcerative colitis; TNFa: anti-tumor necrosis factor α; SAEs: serious adverse events; N/A: not applicable; IV: intravenous; HR: hazard ratio; 95% CI: 95% confidence interval; C_trough_: trough concentration; CRP: C reactive protein; ADAbs: anti-drug antibodies; CDAI: crohn’s disease activity index; TEAEs: treatment-emergent adverse events; C_max_: max concentration; RA: rheumatoid arthritis; AEs: adverse events; FC: fecal calprotectin; SC: subcutaneous; IBD-U: inflammatory bowel disease-unclassified; HBI: Harvey-Bradshaw index; SCCAI: simple clinical colitis activity index.

**Table S2. Clinical trials of adalimumab biosimilars in inflammatory bowel disease**

| **Originator** | **Biosimilar** | **Study design** | **Population** | **Subgroup** | **Efficacy** | **Safety** | **Pharmacokinetics** | **Immunogenicity** | **Reference** |
| --- | --- | --- | --- | --- | --- | --- | --- | --- | --- |
| ADA | ABP 501 and SB5 | Propensity score-weighted, multicenter, cohort study | CD=86  UC=69 | 1. ADA treatment group  2. ABP 501 treatment group  3. SB5 treatment group | The steroid-free clinical remission rates at weeks 8 (40% vs. 50.0% vs. 58.7%) and 32 (49.1% vs. 54.5% vs. 59.0%) between the ADA group, ABP 501 group, and SB5 group were not significantly different. | All AEs in ADA, ABP 501, and SB5 were 3 (3/55, 5.5%), 7 (7/46, 15.2%), and 2 (2/46, 4.3%) | N/A | N/A | (Barberio et al., 2021) |
| ADA | ABP 501 | Multicenter, observational, prospective study | CD= 492  UC=67 | 1. Naïve to ADA and anti-TNFs (group A);  2. Naïve to ADA and exposed to anti-TNFs (group B);  3. Switch from ADA to ABP 501 (group C). | The clinical response rates in patients naïve to ADA was 85.8% (188/219). 165 patients (165/219, 75.3%) achieved a steroid-free remission. | ﻿Total SAEs: 36 (36/559, 6.4%).  SAEs in group A (17.4 person-years) and group B (16.4 per 100 person-years) were higher than group C (4.8 per 100 person-years). | N/A | N/A | (Macaluso et al., 2021a) |
| ADA | ABP 501 | Observational study | CD=87 | 1. ABP 501-start group (naïve to ADA);  2. ABP 501-switch group. | 56% of patients gained clinical remission after ABP 501 treatment. The HBI scores (3.4 vs. 3.8) and CRP levels (4.2 vs. 3.6 mg/L) were not significantly different before and after switching. | AEs: 23 (25.3 %). | N/A | N/A | (Ribaldone et al., 2020) |
| ADA | ABP 501 and SB5 | Multicenter prospective cohort study | CD=88  UC=30 | 1. Switch from ADA to ABP501;  2. Switch from ADA to SB5;  3. Non-switch (ADA treatment) | The remission rates and FC levels were not significantly different before and after switching to ABP 501 (85.4% vs. 76.3%, and 53 vs. 50µg/g, respectively) and SB5 (96% vs. 84%, and 97 vs. 50µg/g, respectively). | N/A | N/A | N/A | (Cingolani et al., 2021) |
| ADA | SB5 | Single, tertiary clinical center, retrospective study | CD=160  UC=24  IBD-U=2 | 1. Switch from ADA to SB5 (SWITCH cohort)  2. ADA maintenance treatment (ORIGINATOR cohort) | The HBI (2 vs 2), PMS (1 vs. 1), CRP levels (1.69 vs. 2.02 mg/L), and FC concentrations (99 vs. 202μg/g) were not significantly different between the SWITCH and ORIGINATOR cohort at Week 10. | The incidence rate of injection pain in the SWITCH cohort (52.7%, 49/93) was higher than the ORIGINATOR cohort (15.1%, 14/93). | C_trough_ levels remained stable before and after switching (14.2 vs. 13.0μg/mL. | ADAbs were same in the SWITCH cohort (2.2%, 2/93) and the ORIGINATOR cohort (2.2%, 2/93). | (Lukas et al., 2020) |
| ADA | SB5 | Observational cohort study | CD=403  UC=60  IBD-U=18 | 1. SB5 switch group  2. SB5 start group | Biochemical remission, fecal biomarker remission, and clinical remission rates were similar before (69.9%, 69.6%, and 82.1%) and at week 26 (70.7%, 58.3%, and 77.5%), and week 52 (70.7%, 59.6%, and 75.4%) after switching. The treatment persistence after 1 year was 62.5% in SB5 start cohort and 83.1% in SB5 switch cohort. | AEs in the switch group: 19.9% (51/256) AEs in the start group: 17.3% (39/225). | The switch group: the C_trough_ levels were stable before and after switching at weeks26 and 52 (10.1 vs. 11.6 vs. 7.8 µg/mL). | The switch group: 7.4% (19/256) patients developed new ADAbs  The start group: 22.0%of patients (40/182) developed new ADAbs. | (Derikx et al., 2021) |
| ADA | SB5 | Prospective, observational study | CD=115  UC=31 | 1. SB5 switch group  2. SB5 start group | The overall remission rates at 12 months were similar in the SB5 start group (60.4%) and the SB5 switch group (74.5%). | AEs: 36.3% (53/146).  The incidence of injection site pain was 24.7% (36/146). | The C_trough_ levels before and at 3 and 6 months after switching were 14.69 µg/mL, 14.99 µg/mL, and 14.31 µg/mL, respectively. | The mean ADAbs levels before and at 3 and 6 months after switching were 15.38mng/mL, 15.51 ng/mL, and 15.29 ng/mL, respectively. | (Tapete et al., 2022) |
| ADA | BI 695501 | Multicenter, randomized, double-blind, phase III trial | CD=147 | 1. BI 695501 treatment group  2. ADA treatment group | The clinical response rates (90% vs. 94% at week 4, and 81% vs. 82% at week 24) and clinical remission rates (68% vs. 75% at week 24) were similar in the BI 695501 and the ADA group. | The incidence of AEs at week 24 in the BI 695501 and the ADA group was 63% and 56%, respectively. | N/A | N/A | (Hanauer et al., 2021) |
| ADA | GP2017 | Multicenter, observational, retrospective study | CD=93  UC=41 | 1. GP2017 treatment group  2. ADA treatment group | The clinical remission rates (82.3% vs. 75.0%) and clinical response rates (87.1% vs. 84.1%) at 12 months were similar in the GP2017 group and the ADA group.  The mucosal healing rate in GP2017 treatment was about 1.5 times as that in ADA treatment (89.2% vs. 60.2%). | The incidence of total AEs was similar between the GP2017 group (1/62, 1.6%) and the ADA group (4/72, 5.6%). | N/A | N/A | (Mocci et al., 2022) |
| ADA | GP2017 | Observational, retrospective study | CD=65  UC=7 | 1. GP2017 start group  2. GP2017 switch group 1 (ADA)  3. GP2017 switch group 2 (ABP 501 and SB5) | 1. GP2017 start group: the remission rates at T6 (10/29, 34.5%) and T12 (17/29, 58.6%) were higher than that at T0 (5/29, 17.2%).  2. GP2017 switch group 1: the remission rates at T0 (25/33, 75.8%), T6 (26/33, 78.8%), and T12 (26/33, 78.8%) were similar.  3. GP2017 switch group 2: the remission rates at T0 (6/10, 60.0%), T6 (7/10, 70.0%), and T12 (7/10, 70.0%) were similar. | AEs: 15.2% (11/72) | N/A | N/A | (Vernero et al., 2023) |
| ADA | FKB327 and GP2017 | Prospective, observational study | CD=34  UC=16 | 1. FKB327 treatment group  2. GP2017 treatment group | The remission or partial response rates in the FKB327 group and the GP2017 group were similar, 81.8% (18/22) and 75.0% (21/27), respectively. | AEs: 14% (7/50) | N/A | N/A | (Wasserbauer et al., 2022) |
| ADA | SB5, APB501, GP2017, and MSB11022 | Retrospective, observational study | CD=371  UC=162 | 1. SB5 treatment group  2. APB501 treatment group  3. GP2017 treatment group  4. MSB11022 treatment group | The clinical remission rates were similar in the SB5 group (161/214, 75.2%), the ABP 501 group (203/259, 78.3%), the GP2017 group (38/49, 77.5%), and the MSB11022 group (9/11, 81.8%). | Total AEs: 6.7%  SB5: 5.1%  APB501: 8.5%  GP2017: 27.3%  MSB11022: 0 | N/A | N/A | (Tursi et al., 2023) |
| ADA | ABP 501, FKB327, SB5, GP2017, and MSB11022 | Observational, retrospective, multicenter study | CD=457  UC=67 | 1. Non-switch cohort (NSC)  2. Switch cohort (SC) | ﻿The cumulative incidence of relapse was similar between the NSC group (3% at 6 months, 6% at 12 months, and 12% at 24 months) and the SC group (3% at 6 months, 6% at 12 months, and 26% at 24 months). | The incidence of AEs in the SC and the NSC was 6% and 5%, respectively. | N/A | N/A | (Casanova et al., 2023) |
| ADA | SB5, APB501, GP2017, and  MSB11022 | Multicenter, retrospective study | CD=127  UC=26 | 1. SB5 treatment group  2. APB501 treatment group  3. GP2017 treatment group  4. MSB11022 treatment group | The clinical remission rates were similar in the SB5 group (51/65, 78.5%), the ABP 501 group (66/78, 84.6%), the GP2017 group (4/7, 66.75), and the MSB11022 group (3/3, 100%) | AEs: 7.9%. | N/A | N/A | (Tursi et al., 2022) |

**Abbreviation**: ADA: adalimumab; CD: crohn’s disease; UC: ulcerative colitis; AEs: adverse events; SAEs: serious adverse events; HBI: Harvey-Bradshaw index; CRP: C reactive protein; FC: fecal calprotectin; IBD-U: inflammatory bowel disease-unclassified; PMS: partial mayo score.

**Reference:**

Barberio, B., Cingolani, L., Canova, C., Barbieri, G., Sablich, R., Urbano, M.T., et al. (2021). A propensity score-weighted comparison between adalimumab originator and its biosimilars, ABP501 and SB5, in inflammatory bowel disease: a multicenter Italian study. *Therap Adv Gastroenterol* 14**,** 17562848211031420. doi: 10.1177/17562848211031420.

Casanova, M.J., Nantes, Ó., Varela, P., Vela-González, M., Rivero, M., Sierra-Gabarda, O., et al. (2023). Real-world outcomes of switching from adalimumab originator to adalimumab biosimilar in patients with inflammatory bowel disease: The ADA-SWITCH study. *Aliment Pharmacol Ther* 58(1)**,** 60-70. doi: 10.1111/apt.17525.

Chaparro, M., Garre, A., Guerra Veloz, M.F., Vázquez Morón, J.M., De Castro, M.L., Leo, E., et al. (2019). Effectiveness and Safety of the Switch from Remicade® to CT-P13 in Patients with Inflammatory Bowel Disease. *J Crohns Colitis* 13(11)**,** 1380-1386. doi: 10.1093/ecco-jcc/jjz070.

Cingolani, L., Barberio, B., Zingone, F., Ferronato, A., Bertani, L., Costa, F., et al. (2021). Adalimumab biosimilars, ABP501 and SB5, are equally effective and safe as adalimumab originator. *Sci Rep* 11(1)**,** 10368. doi: 10.1038/s41598-021-89790-4.

Derikx, L., Dolby, H.W., Plevris, N., Lucaciu, L., Rees, C.S., Lyons, M., et al. (2021). Effectiveness and Safety of Adalimumab Biosimilar SB5 in Inflammatory Bowel Disease: Outcomes in Originator to SB5 Switch, Double Biosimilar Switch and Bio-Naïve SB5 Observational Cohorts. *J Crohns Colitis* 15(12)**,** 2011-2021. doi: 10.1093/ecco-jcc/jjab100.

Fiorino, G., Manetti, N., Armuzzi, A., Orlando, A., Variola, A., Bonovas, S., et al. (2017). The PROSIT-BIO Cohort: A Prospective Observational Study of Patients with Inflammatory Bowel Disease Treated with Infliximab Biosimilar. *Inflamm Bowel Dis* 23(2)**,** 233-243. doi: 10.1097/mib.0000000000000995.

Fischer, S., Cohnen, S., Klenske, E., Schmitt, H., Vitali, F., Hirschmann, S., et al. (2021). Long-term effectiveness, safety and immunogenicity of the biosimilar SB2 in inflammatory bowel disease patients after switching from originator infliximab. *Therap Adv Gastroenterol* 14**,** 1756284820982802. doi: 10.1177/1756284820982802.

Haifer, C., Srinivasan, A., An, Y.K., Picardo, S., van Langenberg, D., Menon, S., et al. (2021). Switching Australian patients with moderate to severe inflammatory bowel disease from originator to biosimilar infliximab: a multicentre, parallel cohort study. *Med J Aust* 214(3)**,** 128-133. doi: 10.5694/mja2.50824.

Hanauer, S., Liedert, B., Balser, S., Brockstedt, E., Moschetti, V., and Schreiber, S. (2021). Safety and efficacy of BI 695501 versus adalimumab reference product in patients with advanced Crohn's disease (VOLTAIRE-CD): a multicentre, randomised, double-blind, phase 3 trial. *Lancet Gastroenterol Hepatol* 6(10)**,** 816-825. doi: 10.1016/s2468-1253(21)00252-1.

Hanzel, J., Jansen, J.M., Ter Steege, R.W.F., Gecse, K.B., and D'Haens, G.R. (2022). Multiple Switches From the Originator Infliximab to Biosimilars Is Effective and Safe in Inflammatory Bowel Disease: A Prospective Multicenter Cohort Study. *Inflamm Bowel Dis* 28(4)**,** 495-501. doi: 10.1093/ibd/izab099.

Jørgensen, K.K., Olsen, I.C., Goll, G.L., Lorentzen, M., Bolstad, N., Haavardsholm, E.A., et al. (2017). Switching from originator infliximab to biosimilar CT-P13 compared with maintained treatment with originator infliximab (NOR-SWITCH): a 52-week, randomised, double-blind, non-inferiority trial. *Lancet* 389(10086)**,** 2304-2316. doi: 10.1016/s0140-6736(17)30068-5.

Luber, R.P., O'Neill, R., Singh, S., Sharma, E., Cunningham, G., Honap, S., et al. (2021). An observational study of switching infliximab biosimilar: no adverse impact on inflammatory bowel disease control or drug levels with first or second switch. *Aliment Pharmacol Ther* 54(5)**,** 678-688. doi: 10.1111/apt.16497.

Lukas, M., Malickova, K., Kolar, M., Bortlik, M., Vasatko, M., Machkova, N., et al. (2020). Switching From Originator Adalimumab to the Biosimilar SB5 in Patients With Inflammatory Bowel Disease: Short-term Experience From a Single Tertiary Clinical Centre. *J Crohns Colitis* 14(7)**,** 915-919. doi: 10.1093/ecco-jcc/jjaa001.

Macaluso, F.S., Cappello, M., Busacca, A., Fries, W., Viola, A., Costantino, G., et al. (2021a). SPOSAB ABP 501: A Sicilian Prospective Observational Study of Patients with Inflammatory Bowel Disease Treated with Adalimumab Biosimilar ABP 501. *J Gastroenterol Hepatol* 36(11)**,** 3041-3049. doi: 10.1111/jgh.15590.

Macaluso, F.S., Fries, W., Viola, A., Centritto, A., Cappello, M., Giuffrida, E., et al. (2021b). The SPOSIB SB2 Sicilian Cohort: Safety and Effectiveness of Infliximab Biosimilar SB2 in Inflammatory Bowel Diseases, Including Multiple Switches. *Inflamm Bowel Dis* 27(2)**,** 182-189. doi: 10.1093/ibd/izaa036.

Massimi, D., Barberio, B., Bertani, L., Costa, F., Ferronato, A., Facchin, S., et al. (2021). Switching from Infliximab Originator to SB2 Biosimilar in Inflammatory Bowel Diseases: A Multicentric Prospective Real-Life Study. *Therap Adv Gastroenterol* 14**,** 17562848211023384. doi: 10.1177/17562848211023384.

Meyer, A., Rudant, J., Drouin, J., Weill, A., Carbonnel, F., and Coste, J. (2019). Effectiveness and Safety of Reference Infliximab and Biosimilar in Crohn Disease: A French Equivalence Study. *Ann Intern Med* 170(2)**,** 99-107. doi: 10.7326/m18-1512.

Mocci, G., Bodini, G., Allegretta, L., Cazzato, A.I., Chiri, S., Aragona, G., et al. (2022). Adalimumab Biosimilar GP2017 versus Adalimumab Originator in Treating Patients with Inflammatory Bowel Diseases: A Real-Life, Multicenter, Observational Study. *Biomedicines* 10(8)**,** 1799. doi: 10.3390/biomedicines10081799.

Reinisch, W., Jang, B.I., Borzan, V., Lahat, A., Pukitis, A., Osipenko, M., et al. (2019). DOP62 A novel formulation of CT-P13 (infliximab biosimilar) for subcutaneous administration: 1-year result from a Phase I open-label randomised controlled trial in patients with active Crohn’s disease. *Journal of Crohn's and Colitis* 13(Supplement_1)**,** S066-S067. doi: 10.1093/ecco-jcc/jjy222.096.

Ribaldone, D.G., Caviglia, G.P., Pellicano, R., Vernero, M., Saracco, G.M., Morino, M., et al. (2020). Effectiveness and safety of adalimumab biosimilar ABP 501 in Crohn's disease: an observational study. *Rev Esp Enferm Dig* 112(3)**,** 195-200. doi: 10.17235/reed.2020.6693/2019.

Schmitz, E.M.H., Boekema, P.J., Straathof, J.W.A., van Renswouw, D.C., Brunsveld, L., Scharnhorst, V., et al. (2018). Switching from infliximab innovator to biosimilar in patients with inflammatory bowel disease: a 12-month multicentre observational prospective cohort study. *Aliment Pharmacol Ther* 47(3)**,** 356-363. doi: 10.1111/apt.14453.

Schreiber, S., Ben-Horin, S., Leszczyszyn, J., Dudkowiak, R., Lahat, A., Gawdis-Wojnarska, B., et al. (2021). Randomized Controlled Trial: Subcutaneous vs Intravenous Infliximab CT-P13 Maintenance in Inflammatory Bowel Disease. *Gastroenterology* 160(7)**,** 2340-2353. doi: 10.1053/j.gastro.2021.02.068.

Smith, P.J., Critchley, L., Storey, D., Gregg, B., Stenson, J., Kneebone, A., et al. (2022). Efficacy and Safety of Elective Switching from Intravenous to Subcutaneous Infliximab [CT-P13]: A Multicentre Cohort Study. *J Crohns Colitis* 16(9)**,** 1436-1446. doi: 10.1093/ecco-jcc/jjac053.

Strik, A.S., van de Vrie, W., Bloemsaat-Minekus, J.P.J., Nurmohamed, M., Bossuyt, P.J.J., Bodelier, A., et al. (2018). Serum concentrations after switching from originator infliximab to the biosimilar CT-P13 in patients with quiescent inflammatory bowel disease (SECURE): an open-label, multicentre, phase 4 non-inferiority trial. *Lancet Gastroenterol Hepatol* 3(6)**,** 404-412. doi: 10.1016/s2468-1253(18)30082-7.

Tapete, G., Bertani, L., Pieraccini, A., Lynch, E.N., Giannotta, M., Morganti, R., et al. (2022). Effectiveness and Safety of Nonmedical Switch From Adalimumab Originator to SB5 Biosimilar in Patients With Inflammatory Bowel Diseases: Twelve-Month Follow-Up From the TABLET Registry. *Inflamm Bowel Dis* 28(1)**,** 62-69. doi: 10.1093/ibd/izab027.

Tursi, A., Mocci, G., Allegretta, L., Aragona, G., Bianco, M.A., Colucci, R., et al. (2023). Comparison of Performances of Adalimumab Biosimilars SB5, ABP501, GP2017, and MSB11022 in Treating Patients with Inflammatory Bowel Diseases: A Real-Life, Multicenter, Observational Study. *Inflamm Bowel Dis* 29(3)**,** 376-383. doi: 10.1093/ibd/izac092.

Tursi, A., Mocci, G., Cuomo, A., Ferronato, A., Elisei, W., Picchio, M., et al. (2022). Replacement of Adalimumab Originator to Adalimumab Biosimilar for a Non-Medical Reason in Patients with Inflammatory Bowel Disease: A Real-life Comparison of Adalimumab Biosimilars Currently Available in Italy. *J Gastrointestin Liver Dis* 31(4)**,** 411-416. doi: 10.15403/jgld-4608.

Vernero, M., Bezzio, C., Ribaldone, D.G., Costa, S., Scalvini, D., Tribocco, E., et al. (2023). Efficacy and Safety of Adalimumab Biosimilar GP2017 in Patients with Inflammatory Bowel Disease. *J Clin Med* 12(21). doi: 10.3390/jcm12216839.

Wasserbauer, M., Hlava, S., Drabek, J., Stovicek, J., Minarikova, P., Nedbalova, L., et al. (2022). Adalimumab biosimilars in the therapy of Crohn´s disease and ulcerative colitis: Prospective multicentric clinical monitoring. *PLoS One* 17(8)**,** e0271299. doi: 10.1371/journal.pone.0271299.

Ye, B.D., Pesegova, M., Alexeeva, O., Osipenko, M., Lahat, A., Dorofeyev, A., et al. (2019). Efficacy and safety of biosimilar CT-P13 compared with originator infliximab in patients with active Crohn's disease: an international, randomised, double-blind, phase 3 non-inferiority study. *Lancet* 393(10182)**,** 1699-1707. doi: 10.1016/s0140-6736(18)32196-2.
